# Phage-induced disturbance of a marine sponge microbiome

**DOI:** 10.1186/s40793-024-00637-7

**Published:** 2024-11-26

**Authors:** Leon X. Steiner, Lara Schmittmann, Tanja Rahn, Tim Lachnit, Martin T. Jahn, Ute Hentschel

**Affiliations:** 1 GEOMAR Helmholtz Centre for Ocean Research Kiel, RD3 Marine Ecology, RU Marine Symbioses, Kiel, Germany; 2 GEOMAR Helmholtz Centre for Ocean Research Kiel, RD1 Ocean Circulation and Climate Dynamics, RU Ocean Dynamics, Kiel, Germany; 3 Zoological Institute, Christian-Albrechts Universität Kiel, Kiel, Germany; 4 Department of Biology, University of Oxford, Oxford, UK; 5 Department of Biochemistry, University of Oxford, Oxford, UK; 6 Christian-Albrechts-Universität Kiel, Kiel, Germany

**Keywords:** Aquatic microbiology, Holobionts, Sponge, Microbiome, Bacteria-virus interactions, Symbionts, Bacteriophages, Temporal dynamics

## Abstract

**Background:**

Bacteriophages are known modulators of community composition and activity in environmental and host-associated microbiomes. However, the impact single phages have on bacterial community dynamics under viral predation, the extent and duration of their effect, are not completely understood. In this study, we combine morphological and genomic characterization of a novel marine phage, isolated from the Baltic sponge *Halichondria panicea*, and report on first attempts of controlled phage-manipulation of natural sponge-associated microbiomes.

**Results:**

We used culture-based and culture-independent (16S rRNA gene amplicon sequencing) methods to investigate bacterial community composition and dynamics in sponge microbiomes with and without the addition of phages. Upon application of a novel Maribacter specialist phage Panino under controlled conditions, we were able to detect community-wide shifts in the microbiome composition and load after 72 h. While bacterial community composition became more dissimilar over time in the presence of phages, species evenness and richness were maintained. Upon phage exposure, we observed the loss of several low-abundance constituent taxa of the resident microbiota, while other originally underrepresented taxa increased. Virulent phages likely induce community-wide disturbances, evident in changes in the total sponge microbial profile by specific elimination of constituent taxa, which leads to an increase in bacterial abundance of opportunistic taxa, such as the genera *Vibrio*, *Pseudoalteromonas*, and *Photobacterium*.

**Conclusions:**

Our findings suggest that sponge microbiome diversity and, by extension, its resilience depend on the maintenance of resident bacterial community members, irrespective of their abundance. Phage-induced disturbances can significantly alter community structure by promoting the growth of opportunistic bacteria like *Vibrio* and shifting the microbiome to a dysbiotic state. These insights highlight the role of bacteriophages in shaping microbiome dynamics and underscore the potential for phage application in managing bacterial community composition in marine host-associated environments.

**Supplementary Information:**

The online version contains supplementary material available at 10.1186/s40793-024-00637-7.

## Background

Bacteriophages, viruses that infect bacteria, exert a significant influence on marine ecosystem functioning and dynamics [1, 2]. The abundance of bacteriophages in seawater is staggering, with estimates reaching several orders of magnitude higher than bacterial counts [3, 4]. In the pelagic zone, bacteriophages orchestrate a delicate balance between bacterial growth and mortality, significantly affecting nutrient cycling and energy flow within marine food webs [5, 6]. This process results in a daily elimination of up to 40% of bacteria, impacting bacterial community composition and function in the marine environment [7]. While lysis reduces the abundance of specific bacterial targets, the integration of phage genomes into bacterial genomes, i.e., prophages, can facilitate the transfer of genetic material [8], enabling the exchange and acquisition of new adaptive traits among different bacteria [9].

More than a decade of research has established organisms as complex ecosystems, where diverse microbes can coexist in complex symbiotic networks with the host, called holobionts or metaorganisms [10–12]. Particularly in corals, one of the best-studied marine animals, phages have long been suspected to influence homeostasis by regulating bacterial populations and maintaining microbial diversity [13–15], protecting mutualistic bacteria [16], and to be essential for holobiont health and resilience [17–19]. Surprisingly, in marine sponges the presence and role of phages have been merely assumed for a long time [20–22], and only later confirmed to host species- [23, 24] and individual-specific viral communities [25]. At the functional level, sponge-associated viruses carry accessory and auxiliary metabolic genes with functions that can improve the resilience and survival of their bacterial hosts [26], such as antimicrobials, defense against toxins [27], and encode genes related to nitrogen metabolism, photosynthesis, and cellulose biosynthesis [28]. Host-associated microbiomes further set the stage for tripartite interactions between the eukaryotic host, microorganisms, and phages. In sponges, bacteria are protected from host phagocytosis by phage-derived ankyrins [25], and in mammals, phages are eliminated by the host immune system [29]. Sponge mediated degradation of phage particles has also been shown to impact the turnover and removal of virions from the water column [30], and host tissues [31]. As phages are key modulators of bacterial communities, both in seawater and marine animals, one does wonder how the effects of individual phages contribute to the dynamics of microbiota changes.

The effects of single phages on bacterial communities in vivo have been extensively studied with the application of phage therapy, mostly in the context of disease [32]. Such investigations focus on the targeted reduction of pathogenic, often multidrug-resistant, bacteria [33]. However, these studies often do not address the broader alterations in the resident bacterial community [34]. When such studies do explore community-wide changes, their focus usually lies on potential collateral effects of the therapy on residual microbiome members in terms of general community composition at the time of application [35, 36]. The controlled application of phage therapy in complex marine environments and its successful application in marine animals, have also been demonstrated on bacterial diseases in corals [37–41]. However, simplified animal model systems, e.g. mice with reduced microbiome complexity, are needed to extensively study the fundamental interactions of individual phages with their bacterial hosts in microbiomes [42]. While they demonstrated that individual interactions lead to promotion or regression of single bacterial taxa outside of the phage’s infection network, cascading effects that result in changes in the entire bacterial community have also been observed [43, 44]. However, this brings up the question if these phage-induced effects scale with the size of a phage infection network and abundance of bacterial hosts.

Sponges as filter-feeding organisms and their microbial symbionts are continuously exposed to the microbial diversity of seawater [30]. Despite the constant influx of environmental bacteria and viruses, sponges are able to maintain a diverse and specific microbial community [45]. Sponge microbiomes have demonstrated a high degree of functional redundancy [46] and are impervious to changes by environmental stressors [47]. Compositional stability is also evident at different taxonomic layers, but has been shown to vary between different sponge species, attributing their functional resilience to the ability to restructure their microbiomes with horizontal transmission [48]. In general, microbiome perturbations in seawater by environmental stressors have been shown to induce short-term oscillations which ultimately have no impact on the ecological resilience of bacterial communities in the long-term [49]. While the short-term maintenance of a high microbial diversity may not be relevant for sponge microbiome functioning, higher diversity in host-associated microbiomes has been shown to protect against pathogen invasions by nutrient blocking [50]. Experimental perturbations of the sponge microbiome with antibiotics have shown that while these disturbances wane over time and return to their initial state, opportunistic colonizers and facultative pathogens can induce dysbiotic states [51].

Making use of the low microbial abundance (LMA) sponge species *Halichondria panicea* [52], previously established in animal-microbe symbioses research [53], we aimed to isolate effects of a single phage on sponge-associated bacterial communities. We chose a culturable bacterial sponge symbiont of the genus *Maribacter* [54] as the target for phage isolation and manipulation within the sponge microbiome. Due to the LMA status of the sponge microbiome, being dominated (> 50% relative abundance) by a single unculturable symbiont *Ca.* Halichondribacter symbioticus, remaining microbiota each fluctuate around < 1% relative abundance in natural, aquarium, and experimental sterile conditions [51, 53]. With the application of a single highly specialized phage, this allows us to investigate the importance and impact of low abundant taxa on dynamics of complex bacterial communities.

Our null hypothesis was that in a diverse bacterial community such as the sponge microbiome, after a single species loss event, no significant changes in the diversity of the sponge microbial community would be detected due to the functionally redundant property of stable and resilient host-associated microbiomes [55]. With this goal, we characterized changes in the sponge *H. panicea* microbiome subjected to either direct injection of phage particles into sponge tissue or suspension into surrounding seawater, combined with high and low doses. We followed the microbiome 16S taxonomic profile in sponge tissues over time to assess the degree of community changes, and measured changes in bacterial load of total culturable microbiota. In particular, we looked for sponge-associated bacterial taxa able to proliferate due to the disruption of the microbiome due to specific phage predation.

## Results

### Characterization of the novel Maribacter phage Panino

**Fig. 1:**
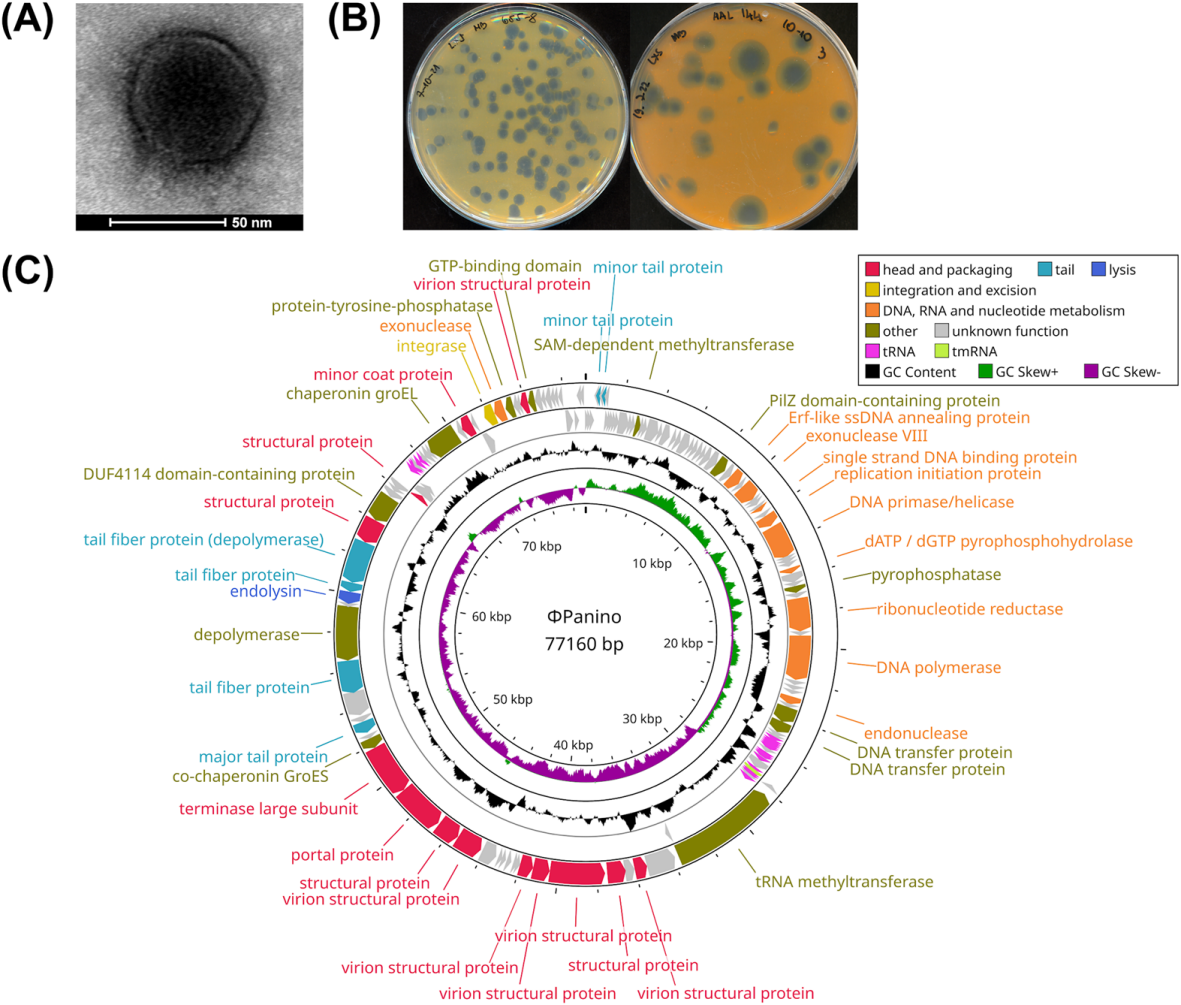
Morphological and genomic representation of Maribacter phage Panino. **A** Transmission electron microscopy photograph showing Maribacter phage virions with short, noncontractile tails, characteristic of the podoviral morphology. **B** Representative images of clear phage plaques after 48 h (left) and turbid halo development after 5 days (right) of incubation on M. halichondriae bacterial lawns. **C** Circular genome map of Maribacter phage Panino. The two outer rings show ORFs and tRNA genes, based on the direction of transcription, and colored by functional categories; two inner rings show the histogram distribution of GC content (black) and GC skew (green, purple)

Transmission electron microscopy (TEM) revealed an icosahedral capsid with a diameter of ∼60 nm and a short noncontractile tail of ∼15 nm, which is a characteristic feature of podoviral phages (Fig. 1A). Clear plaques with turbid halos formed on a *M. halichondriae* lawn grown on MB agar plates at 23 °C after 48 h (Fig. 1B, Fig. S1A). Halos were observed after 5 days and increased in size with further incubation, indicative of phage-encoded polysaccharide depolymerase activity (Fig. 1B). The phage, named Panino, consisted of linear double-stranded DNA with a 77,160 bp genome and an average GC content of 37.73% and coding density of 93.26% (Fig. 1C). The genome contained a total of 132 predicted open reading frames (ORFs). Interestingly, about half of those ORFs (63/132) were located on the reverse strand, suggesting a genomic organization in two clusters. The ORFs on the positive strand encode functions related to nucleotide metabolism and genome replication, while the reverse strand encodes phage structural, lysis, and integration-related functions. One-third (43/132) of predicted genes showed similarity to previously defined functions, while the remainder (89/132) was annotated as hypothetical proteins with unknown function (Table S1) and spurious hits in public databases. Out of the 43 ORFs with defined functions, 19 encoded head and tail structural proteins, e.g., phage terminase large subunit, major/minor tail and tail fiber proteins, portal and coat protein, and other likely capsid-related virion structural proteins comprising the structure and packaging module of the phage genome. The nucleotide metabolism and genome replication module included 10 genes, i.e., those for exo-/endonuclease activity, DNA polymerase and primase/helicase, ribonucleotide reductase, replication initiation, and Erf-like ssDNA annealing protein; involved in the circularization of the linear dsDNA phage genome upon entry into the host cell [56]. The lysis module was represented by one endolysin gene, an N-acetylmuramoyl-L-alanine amidase, an enzyme that specifically cleaves covalent bonds of the cell wall peptidoglycan causing cell lysis. We found both phage-specific chaperonin GroEL and GroES homologs, as well as pyrophosphatase, transfer, DUF4114-domain and PilZ-domain, and depolymerase proteins with hydrolase activity. We found a total of 20 tRNAs within our phage genome, ranking it at the 86th percentile in count and 96th percentile based on tRNA to genome size ratio, when compared to all other published *Caudoviricetes* phages (Fig. S1B). Additionally, we identified one transfer-messenger RNA (SsrA) gene, but no proteins with homologies to RepL or ankyrin-repeat, no known antiCRISPR proteins, or proteins encoded by biosynthetic gene clusters.

**Fig. 2:**
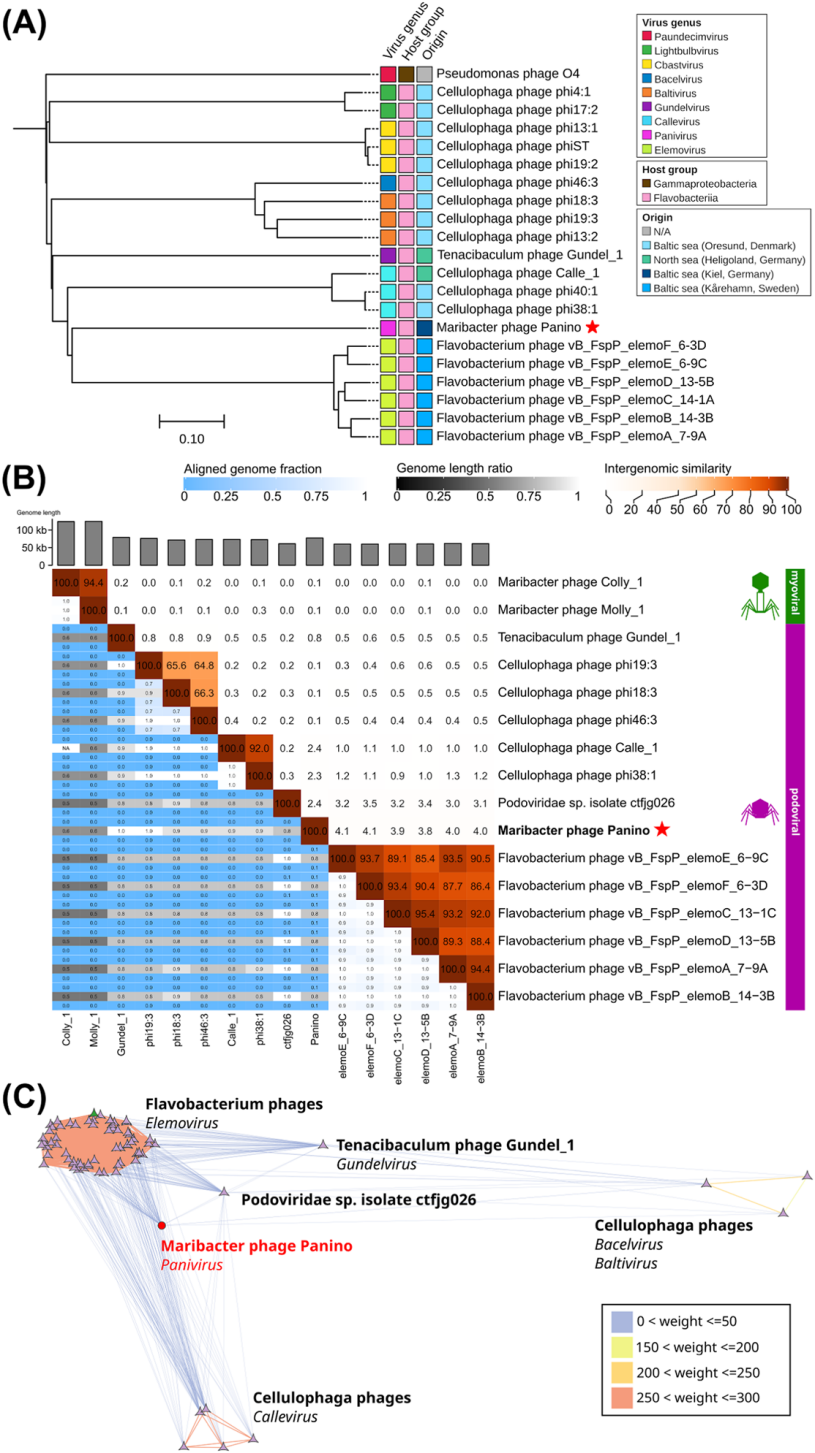
Phylogenetic relatedness and genomic similarity of Maribacter phage Panino to other related phages. **A** Vip-Tree generated phage proteomic tree of Maribacter phage Panino and 20 other closest related dsDNA viruses calculated by BIONJ based on genomic distance matrices, shown by linearly scaled branch length values of normalized tBLASTx scores. Novel sponge phage Panino indicated by a red star. **B** Genome similarity heatmap of closely related reference phages from the North and Baltic Sea. On and above the diagonal, the color-coding (red) indicates the clustering of the phage genomes based on intergenomic similarity and values represent the similarity for each genome pair. Below the diagonal, colors (blue and gray) and values show fractions of aligned genomes that show similarity. Novel sponge phage Panino indicated by a red star. **C** Visualized vConTACT2 analysis of shared gene subnetwork with ICTV recognized bacteriophage genera of related phages (nodes) infecting other flavobacterial hosts. Edge color and width proportional to its weight

The genome similarity algorithm of ViPTree suggested genomic similarity (S_G_) scores of less than 0.1 for phage Panino with any other phage from the reference database (Table S2). Furthermore, we constructed a non-rooted proteome tree from the closest 20 phages (Fig. 2A). Phage Panino clustered separately from other podoviral *Flavobacterium* and *Cellulophaga* phages. Intergenomic distance calculations by VIRIDIC (Fig. 2B) with 13 publicly available phage genomes identified by network analysis (Fig. 2C), revealed that phage Panino is < 5% similar to aforementioned *Flavobacterium* phages and < 3% similar to *Cellulophaga* phages. We included two Maribacter phage genomes previously isolated from the North Sea [57] as the only other phages to date isolated from the same host genus, albeit being myoviruses and < 1% similar to phage Panino. Based on our polyphasic taxonomic assessment, from the phylogenetic analysis, and intergenomic distance calculations, it is clear that phage Panino represents a novel phage establishing a new phage genus. According to the recommendations set by the International Committee on Taxonomy of Viruses (ICTV) [58], we propose the establishment of a new viral genus *Panivirus*, with phage Panino as the only member and main representative.

**Fig. 3:**
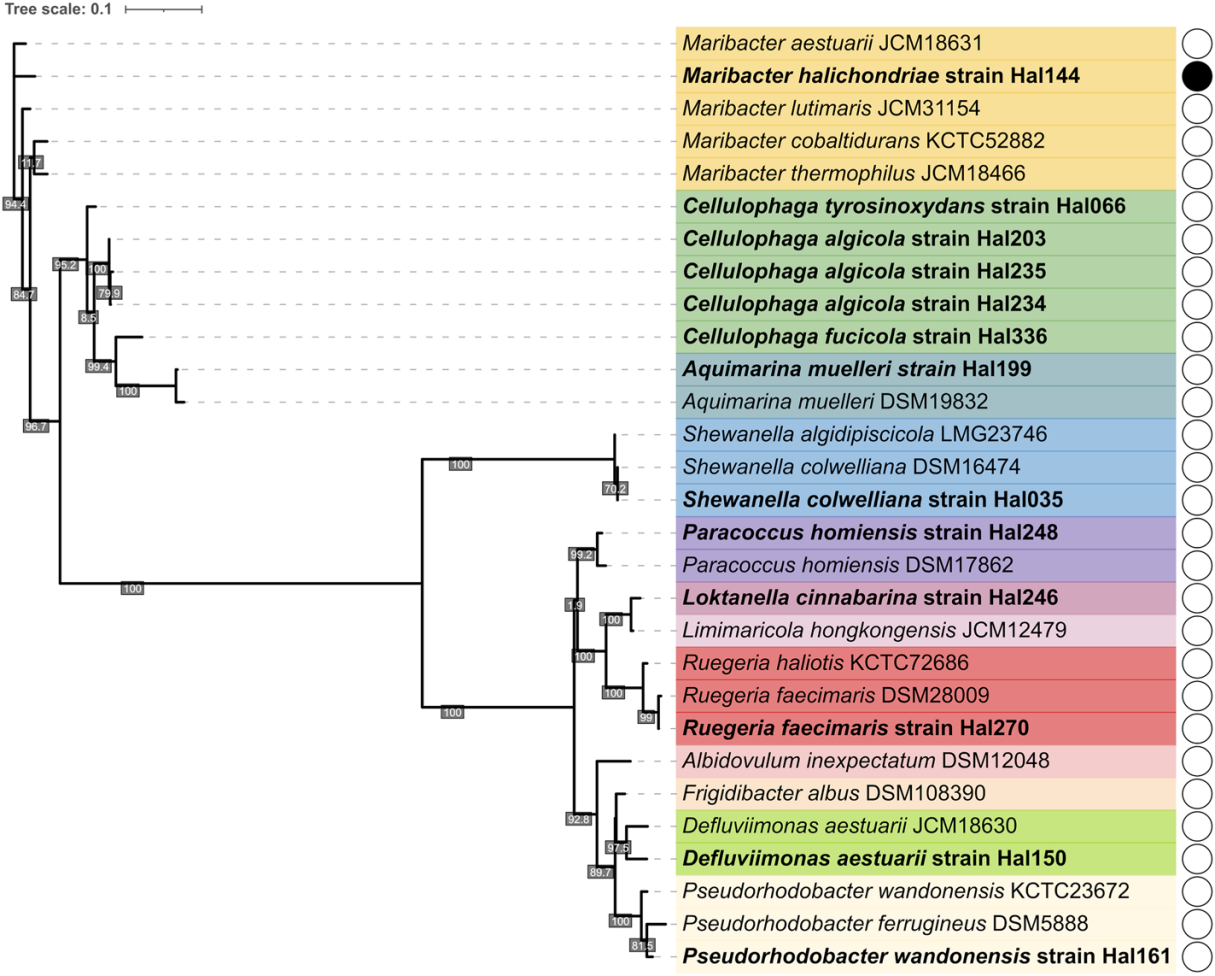
Phage host range test with sponge-associated and reference bacterial type strains. Maximum-likelihood phylogenetic tree of 16S rRNA gene sequences of a subset of tested bacterial isolates for Maribacter phage Panino infectivity. Each genus is marked by a different color, bacterial strains isolated from *H. panicea* sponge tissue indicated in bold, bacterial strains from public culture collections show their corresponding ID in the name. Results of phage host range tests shown next to each bacterial strain indicated by a full (original propagation host, bacterial lysis) and empty circle (no lysis)

We evaluated the host specificity by using a total of 73 strains of different genera and species within Flavobacteriales, related to *M. halichondriae* (Table S3) from public (16/73), and private (57/73) strain collections isolated from the same sponge. We observed that phage Panino was highly specific in its infection activity and did not show any signs of productive or non-productive lysis on any other tested strain except *M. halichondriae* (Fig. 3).

### Phage-induced changes of bacterial load

**Fig. 4:**
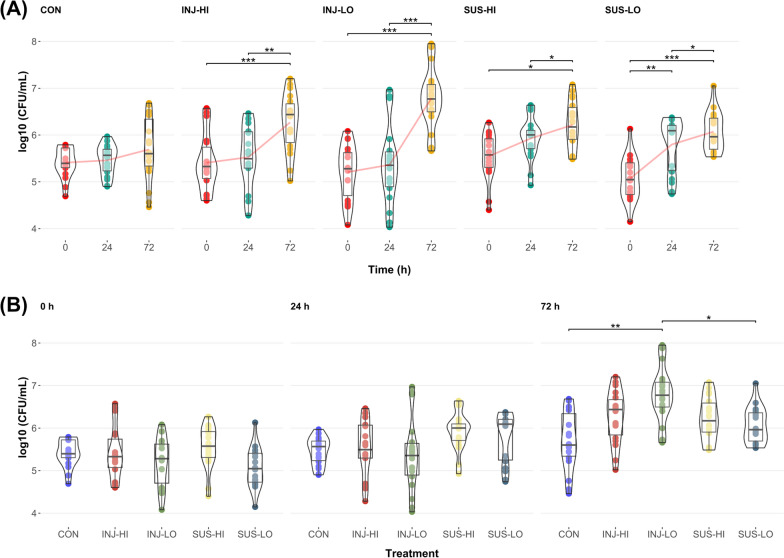
Total culturable bacterial abundance in sponge tissue in control (n = 18) and phage treatments (n = 18) over time. **A** Boxplot comparison within treatments—control treatment (CON), injection into sponge tissue with high (INJ-HI) and low phage concentration (INJ-LO), suspension into seawater of high (SUS-HI) and low phage concentration (SUS-LO) treatments. Red lines connect means in a series. Data analyzed with one-way repeated measures ANOVA, FDR adjusted with Holm’s method, post-hoc pairwise comparisons with the Student’s *t*-test; significant comparisons indicated as * <0.05, ** <0.01, *** <0.001. **B** Boxplot comparison between treatments at 0, 24, and 72 h. Data analyzed with Welch's one-way ANOVA, FDR adjusted with Holm’s method, post-hoc pairwise comparisons with the Games-Howell test; significant comparisons indicated as * <0.05, ** <0.01, *** <0.001

To test the effect of phage Panino on sponge-associated bacteria, we conducted an experiment where we monitored the change in bacterial community composition and abundance over 72 h. Purified phage particles were either injected directly into the sponge tissue or suspended into the surrounding seawater in a controlled experimental setting. These were also performed in combination with a highly concentrated phage titer and a lower dilution to test whether the strength of the response is dose-dependent as well. Sponge tissue samples (n = 6) from each treatment were taken at 0, 24, 72 h, and plated for total culturable bacteria (CFU) in triplicates (n = 18). Both the effects of time and treatment had a significant effect on the culturable abundance (ANOVA, F(8, 255) = 5.22, *p* = 4.63E-06), although the stronger effect was due to time (ANOVA, F(2, 255) = 56.01, *p* < 2.00E-16) whereas the effect of treatment was approximately 16 times lower (ANOVA, F(4, 255) = 3.38, *p* = 0.01) (Table S4). Within each phage treatment we could observe a significant increase in bacterial load over time, based on a linear regression model the largest increase was evident in the phage treatment INJ-LO (β = 0.023, *p* < 1.00E-04), and the smallest in SUS-HI (β = 0.009, *p* = 2.36E-04), while the control exhibited a non-significant positive relationship (β = 0.004, *p* = 0.07) (Table S4). Comparison of changes within each sponge individual of a treatment (0–24 h, 24–72 h, 0–72 h; one-way repeated measures ANOVA, post-hoc Student’s *t*-test, *p* < 0.05), displayed a significant increase in abundance after each time point, whereas the CFU increase in the control was not statistically significant (Fig. 4A, Table S5). In the injection treatments, regardless of phage concentration, we could observe significant changes from 0 to 72 h, and from 24 to 72 h. In the suspension treatments, the significant differences were also from 0 to 72 h and 24 to 72 h, with the low concentration treatment also showing a significant difference from 0 to 24 h. Comparison between control and phage treatments at individual time points only indicated a significantly different CFU count in the INJ-LO treatment compared to the CON and SUS-LO treatment at 72 h (Welch's one-way ANOVA, Games–Howell post-hoc test, *p* < 0.05) (Fig. 4B, Table S5). We also used a linear mixed model to adjust for inherent variability between sponge individuals to ensure that observed effects of phage treatments on culturable abundance were not solely due to sponge individual differences, which confirmed the significant increases with time in phage treatments, and differences between the aforementioned injection and suspension treatments at 72 h (Table S4). Based on these findings, while all phage treatments induced an increase in bacterial abundance over time, the injection treatments showed the most pronounced effect.

### Phage-induced changes in bacterial community composition

**Fig. 5:**
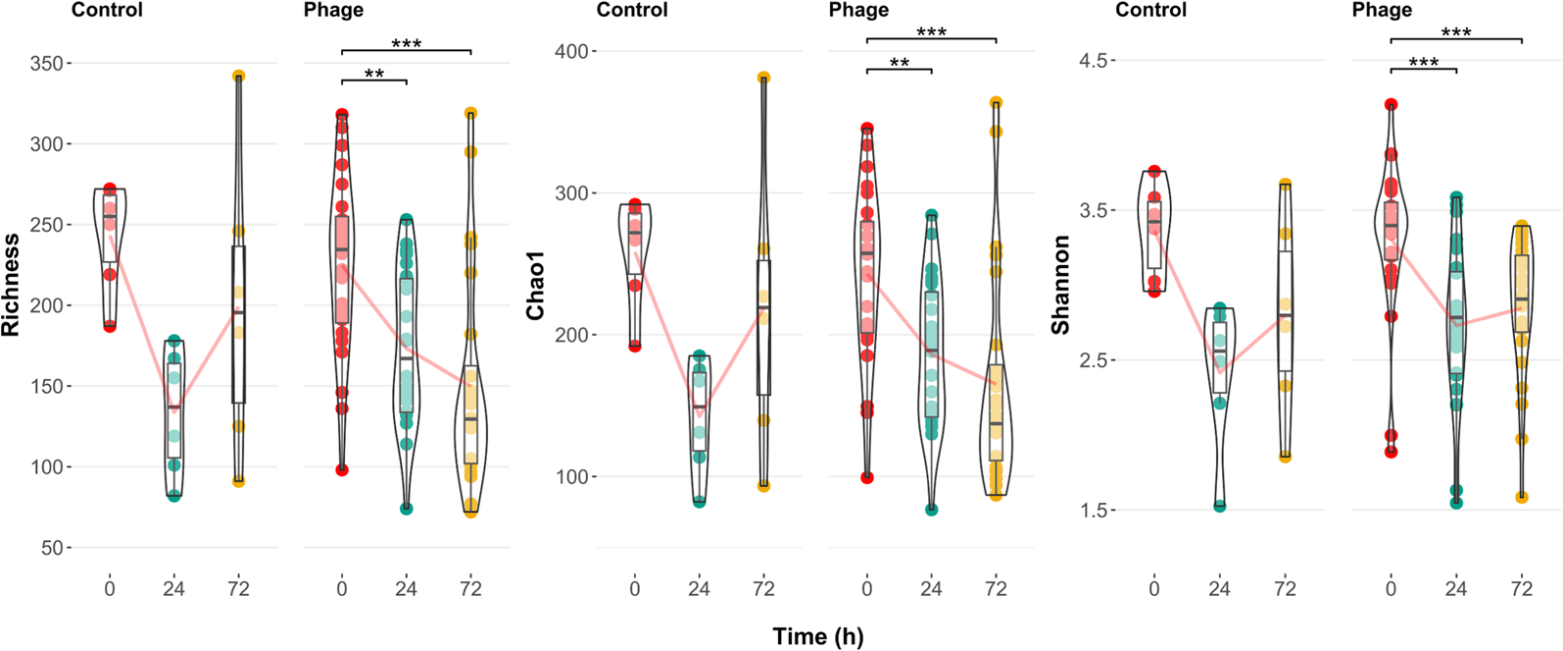
Alpha diversity measurement in sponge tissue in control (n = 6) and phage treatments (n = 24) over time. Boxplot comparison of species richness (observed count, Chao1, and Shannon’s diversity index) between each time point (0, 24, 72 h) for control and phage-treated samples. Data analyzed with a Friedman rank sum test, Durbin–Conover post-hoc test; significant comparisons indicated as * <0.05, ** <0.01, *** <0.001

In order to assess changes in composition and relative abundance, we profiled the bacterial community with 16S rRNA gene sequencing. To study the overall effects of phages on the sponge microbiome, we pooled samples of individual phage application methods and compared them to the control for further analysis, since the individual differences between them were negligible throughout the experiment (Tables S6, S9). The within sample diversity (alpha diversity) (Fig. 5, Table S7) significantly decreased with phage application over time, in terms of richness and evenness. In the control, alpha diversity decreased from 0 to 24 h but rebounded at 72 h, with the overall changes not being statistically significant. However, no significant differences in alpha diversity were observed between the control and phage treatment at all time points (Fig. S3, Table S7).

**Fig. 6:**
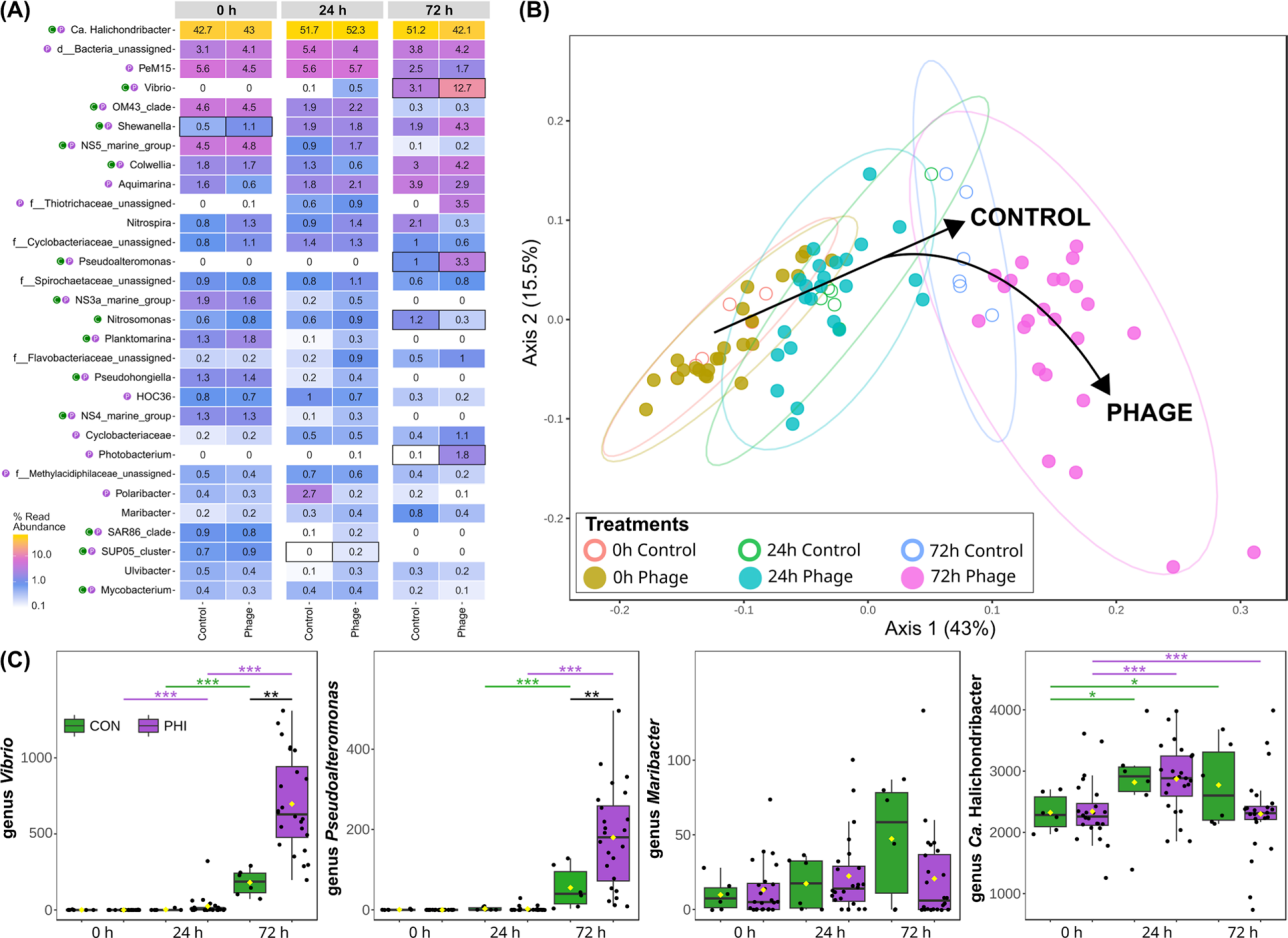
Compositional changes of the bacterial community in sponge tissue in control (n = 6) and phage treatments (n = 24) over time. **A** Relative taxonomic abundance of top 30 abundant taxa at the genus level for pooled samples in control and phage treatments at 0, 24, and 72 h. Differentially abundant taxa that change with time are indicated in the taxon names for the control and phage, and with a black box for differential abundance between treatments. **B** Beta diversity changes shown with a principal coordinate analysis (PCoA) based on Jensen﻿–Shannon distance (PERMANOVA F-value: 13.898; *p*-value: 0.001) showing dissimilarity between ASV compositions in sponge tissue samples during 72 h. Treatments are depicted using different symbols (control—empty circle, phage—full circle), colors indicating three time points and grouped by ellipses (95% confidence). These two components explain 58.5% variance, the overall bacterial community composition (beta diversity) changed from the start to the end of the experiment, with control and phage treated samples diverging after 72 h. **C** Read abundance counts of selected bacterial taxa tested for differential abundance with a Multivariable Association with Linear Models (MaAsLin2) test in control (CON) and phage (PHI) treatments. Significant differences indicated with false discovery rate (FDR) as * <0.05, ** <0.01, *** <0.001. Differences between 0 and 72 h in CON and PHI treatments for the genus *Vibrio* and *Pseudoalteromonas* were significant but omitted from the figure for simplicity and are listed in Table S13

We did not observe larger compositional differences at 0 and 24 h between the treatments, with the main symbiont, *Ca.* Halichondribacter symbioticus always dominating the bacterial profile of the sponge (Fig. 6A). ASVs assigned to *Ca.* Halichondribacter increased in relative abundance in both control and phage treatments from 0 to 24 h (43% to 54% for control, 43% to 53% for phage), while groups of ubiquitous aquatic bacteria decreased in relative abundance, such as NS5 (4.8% to 0.8% for control, 4.8% to 1.7% for phage), NS3a (1.9% to 0.2% for control, 1.6% to 0.4% for phage) and NS4 marine group (1.3% to 0.1% for control, 1.3% to 0.3% for phage), OM43 (4.4% to 2% for control, 4.5% to 2.2% for phage), and SAR86 clade (0.9% to 0.1% for control, 0.8% to 0.2% for phage). Other taxa from such ubiquitous groups followed a similar trend albeit towards the end of the experiment from 24 to 72 h, like PeM15 (5.8% to 2.5% for control, 5.7% to 1.7% for phage) and HOC36 (0.9% to 0.2% for control, 0.7% to 0.2% for phage). Changes became larger at 72 h, taxa of the genus *Vibrio* increased in relative abundance in the phage treatment (0.4% to 13%) compared to the control (0.04% to 3%), while taxa of *Ca.* Halichondribacter decreased more in the phage treatment (53% to 42%) compared to the control (54% to 52%) (Table S8).

We compared phage-induced changes in bacterial composition and between sample diversity (beta diversity) of phage-treated and control samples. We performed a Principal Coordinate Analysis (PCoA) based on Jensen﻿–Shannon divergence distances (Fig. 6B). While we observed significant differences between each time point within treatments, there were no differences between the phage and the control treatment at 0 h (PERMANOVA, F-value = 0.54, FDR = 0.65) and 24 h (F-value = 0.98, FDR = 0.42), even with Bray–Curtis, Jaccard, weighted and unweighted Unifrac diversity measures. However, at 72 h, we observed a significant change in bacterial composition in the phage treatment compared to the control (PERMANOVA, F-value = 5.02, FDR = 0.01) (Fig. 6A, Table S10), which was also observed for individual phage treatments (Fig. S4, Table S9). Phage-treated samples displayed more divergence as the effect of phage treatment on Jensen﻿–Shannon dissimilarity was larger (approximately 3.8 times) between 0 and 72 h (PERMANOVA F-value = 82.76, FDR = 0.002), compared to in the control (F-value = 21.83, FDR = 0.01) (Table S10). Axis 1, explaining 43% of the change, corresponded with the experimental timeline (R^2^ = 0.87, F = 574.70, *p* < 2.20E-16) (Fig. 6B).

### Phage-induced changes in relative abundance of selected bacterial lineages

With an LDA effect size analysis, we could identify several other genera as significant features that responded to the phage treatment (Fig. S5, Table S11). One of the top discriminant and significant features (FDR < 0.05, LDA > 2.00, 94/320) of the phage treatments was the genus *Vibrio* that increased in abundance over the course of the experiment (Fig. 6C), based on single-factor univariate (FDR = 1.96E-12, H-statistic = 74.31) (Table S12) and multiple linear regression analyses (Table S13). The largest increase was evident between 0 and 72 h (Log2FC = 9.76, SE = 0.42, FDR = 4.28E-27) and 24 to 72 h in phage treatments (Log2FC = 7.59, SE = 0.42, FDR = 5.30E-22), which were also larger than their respective changes in control between 0 and 72 h (Log2FC = 7.01, SE = 0.85, FDR = 2.59E-09) and 24 and 72 h (Log2FC = 6.46, SE = 0.85, FDR = 5.26E-08). After 72 h the abundance of *Vibrio* was also approximately 5 times higher in the phage treatment than in the control (Log2FC = 2.51, SE = 0.67, FDR = 4.14E-03). Additionally, the effect of phage treatment was evident with a faster onset time of *Vibrio*, between 0 and 24 h, which wasn’t detected in the control (Log2FC = 2.17, SE = 0.42, FDR = 4.13E-05).

In total, more differentially abundant features were detected between respective time points in the phage treatment (0–24 h: 69; 24–72 h: 69; 0–72 h: 111) than in the control (0–24 h: 52; 24–72 h: 21; 0–72 h: 57) (Table S13). Within each treatment, the proportion of features with a positive change was higher in the control (0–24 h: 15.38%; 24–72 h: 14.04%; 0–72 h: 42.86%) than in the phage treatments (0–24 h: 13.04%; 24–72 h: 23.42%; 0–72 h: 26.09%). Between 24 and 72 h, only 24.63% of significantly different features in the phage treatments were also detected to account for 89.95% features in the control. Comparing the abundance at the beginning and the end of the experiment (0 to 72 h), the number of shared features with differential abundance between the treatments increased to 48.64% for the phage treatments and 94.73% for the control. The number of differentially abundant features between the control and the phage treatments increased at each time point (0 h: 2; 24 h: 4; 72 h: 16).

In addition to *Vibrio*, we detected significant abundance changes in 15 other genera between the control and the phage treatments at 72 h (Table S13). Most of them (11/15) belonged to different orders of Gammaproteobacteria, namely Alteromonadales, Burkholderiales, Oceanospirillales and Vibrionales, in addition to several uncharacterized bacterial groups such as KI89A clade, UBA10353 marine group, and Ga0077536. The abundance change in the genus *Photobacterium* between control and phage treatments at 72 h, was higher than all other 15 detected features (Log2FC = 5.29, SE = 0.65, FDR = 4.42E-10) as it wasn’t found in the control. However, in addition to *Vibrio*, we found the genus *Pseudoalteromonas* (FDR = 1.96E-12, H-statistic = 74.08) to follow a similar profile with significant abundance changes in phage treatments (Fig. 6C). The largest increase was also between 0 and 72 h (Log2FC = 7.38, SE = 0.35, FDR = 1.63E-25) and 24 to 72 h in phage treatments (Log2FC = 6.93, SE = 0.35, FDR = 7.50E-24), which were larger than their respective changes in control between 0 and 72 h (Log2FC = 4.79, SE = 0.70, FDR = 2.66E-07) and 24 and 72 h (Log2FC = 4.17, SE = 0.70, FDR = 1.39E-05) as well. Similarly, after 72 h the abundance of *Pseudoalteromonas* was also higher in the phage treatment than in the control (Log2FC = 2.37, SE = 0.57, FDR = 1.05E-03).

To assess the impact of the Panino phage on its bacterial sponge symbiont host *M. halichondriae*, we followed the abundance of the genus *Maribacter* over time (Fig. 6C). Even though the observed abundance changes in count data were not significant (FDR = 0.63, H-statistic = 4.96) (Table S12) or detected as differentially abundant features, the combined relative abundance of ASVs assigned to this genus remained lower in the phage treatment than in the control (control 0.89% vs. phage 0.34%) (Table S8). This was particularly evident between 24 and 72 h, when the control showed an increase (0.26% to 0.89%) while the phage treatment did not (0.39% to 0.34%).

Further differences could be detected in the abundance of the main symbiont genus *Ca.* Halichondribacter (Fig. 6C). While the abundance changes between 0 and 24 h in control (Log2FC = 0.95, SE = 0.31, FDR = 1.87E-02) and phage treatments (Log2FC = 0.62, SE = 0.15, FDR = 9.93E-04) were significantly different, there was no further change between later time points (Table S13). Furthermore, no differentially abundant features within the genus were found between the control and phage treatments at each respective time point.

Within top 20 highest ranking discriminant features at the ASV level, between the control (n = 6) and phage treatments (n = 24) during the course of the experiment identified by an LDA effect size analysis (LDA > 2.00, FDR > 0.05) (Table S11), we found the five ASVs of the genus *Vibrio*. We identified the two most abundant ASVs to classify as *V. lentus* (100% identity) or *V. cyclitrophicus* (100%), and *V. splendidus* (99.26%) based on their best scoring BLAST hits against the NCBI 16S rRNA (Bacteria and Archaea type strains) database (Table S14).

### Microbiome diversity changes and dysbiosis

We assessed changes in community diversity in terms of a perturbed or altered bacterial community state by calculating dysbiosis scores for each treatment at 0, 24, and 72 h. Dysbiosis was defined as the difference in Euclidean distance for a sample to the treatment group centroids, using Bray–Curtis differences, and control samples as the reference group. The thresholds for dysbiosis and normobiosis, based on scores of all samples at 0 h when no effect of treatment or time was evident, were 0.015 and − 0.008. A value of zero indicates that a sample lies at equal distance from the centroids of both classes (control and treatment), while higher dysbiosis scores indicate greater deviation from normobiosis. At the initial time point, the control group (CON) had a mean score of − 0.008 (SD = 0.004), while the phage-treated groups exhibited higher mean scores INJ-HI: 0.004 (SD = 0.003), INJ-LO: 0.010 (SD = 0.005), SUS-HI: 0.008 (SD = 0.006), SUS-LO: 0.007 (SD = 0.007). Only one sample from the INJ-LO, SUS-HI, and SUS-LO treatments was above the dysbiosis threshold at this time point, although all differences and scores were generally low (< 0.01).

At 24 h, the control group remained in a normobiotic state with a mean score of − 0.016 (SD = 0.045). However, all phage-treated groups showed an increase in dysbiosis scores—INJ-HI: 0.017 (SD = 0.006), INJ-LO: 0.018 (SD = 0.007), SUS-HI: 0.016 (SD = 0.007), SUS-LO: 0.014 (SD = 0.010). The number of samples classified as dysbiotic increased, with 3/6 in INJ-HI, 5/6 in INJ-LO, 4/6 in SUS-HI, and 4/6 in SUS-LO, with a total of 16/24 phage-treated samples marked dysbiotic.

By 72 h, the control group's mean score further decreased to − 0.060 (SD = 0.023), consistent with a normobiotic state. In contrast, the phage-treated groups indicated a further increase in dysbiosis scores—INJ-HI: 0.068 (SD = 0.035), INJ-LO: 0.090 (SD = 0.096), SUS-HI: 0.046 (SD = 0.061), SUS-LO: 0.035 (SD = 0.034). All samples in the INJ-HI group (6/6) were classified as dysbiotic, along with 4/6 in INJ-LO, 4/6 in SUS-HI, and 5/6 in SUS-LO, yielding a total of 19/24 phage-treated samples detected as dysbiotic at this time point.

## Discussion

### A novel Maribacter phage with a narrow host range to infect a bacterial sponge symbiont

This is the first study to isolate phages from the sponge *Halichondria panicea*, shown to specifically infect a single symbiont and modulate the sponge-associated bacterial community. The isolated Maribacter phage Panino was shown to infect the recently described sponge symbiont *M. halichondriae*. Even though the phage was isolated from viral particles enriched in sponge tissues, principally it could also be present in seawater due to the filter-feeding nature of sponges. The genus *Maribacter* is known to be globally present in the water column and other marine organisms [54, 59], and distantly related myoviral Maribacter phages have been identified in the North Sea [57]. In addition to this study, evidence of sponge-associated phages and their description has been scarce so far but with few exceptions of sipho- and myoviral phages [60, 61]. Only one study to date has employed experimental manipulation of the siphoviral phage Tedan, from the sponge *T. anhelans*, that demonstrated to infect and influence its sponge bacterial symbiont *Ruegeria*, to be better adapted to the sponge microenvironment [62].

Due to the inability of this novel Maribacter phage Panino to infect several other closely and distantly related bacterial hosts from the same sponge or other habitats, it seems likely to belong to a group of phages endemic to the sponge. This inability to infect is in line with findings suggesting geographic and niche separation of bacterial strains as one of the determinants of phage host tropism [63]. Even though phages are more likely to infect bacteria originating from the same site [64, 65], we saw no signs of productive lysis on other *Maribacter* strains obtained from the same sponge isolation source (Fig. 3).

Despite testing the susceptibility of other potential hosts—sponge symbiont bacteria of the same genus infected by related Baltic and North Sea phages and their publicly available related reference bacterial strains—no evidence of a productive lysis except on the original propagation host was observed. Phage host range has been shown to be determined by the lifestyle of the bacterial host, with a higher degree of specialization happening in systems with bacterial cross-feeding and trophic dependency [66]. Since phage Panino was unable to lyse other tested bacterial strains, it is possible that its bacterial host *M. halichondriae* displays a high degree of specialization and trophic dependency in the sponge microbiome.

Sequence-based comparisons with publicly available viruses confirmed the phage as a novel viral genus within the *Caudoviricetes* class, with phage Panino as its sole known representative. The Maribacter phage Panino showed common morphological features (Fig. 1A) with other related tailed podoviral phage families. It largely diverged from related *Cellulophaga* and *Flavobacterium* phages based on proteome phylogenetic tree, genome similarity clustering, and network analyses (Fig. 2). The only other Maribacter phages described to date are myoviral-like and show little to no similarity to the phage Panino genome.

In terms of functional properties, phage Panino showed no sensitivity to chloroform, implying an absence of a lipid membrane, displayed constitutive virulence and a virulent lifestyle despite the presence of an integrase. The high virulence could be attributed to the presence of a high number of tRNA genes (Fig. 1C, Fig. S1B), which falls in line with previous findings that the presence of tRNAs is a characteristic of highly virulent phages [67]. Based on the narrow host range, low similarity with phages infecting other bacterial genera, and large tRNA gene repertoire—we postulate this novel phage to have likely originated from another, likely free-living, flavobacterial host and recently adapted to a specific sponge bacterial symbiont acquiring the corresponding bacterial tRNA genes after a successful lysis event.

Based on the detection of an integrase gene in the phage genome (Fig. 1C), a temperate lifestyle with the integration into the host genomic DNA could be assumed. Additionally, tRNA and tmRNA serve as attachment sites for integration of phages into the bacterial host genome [68], which phage Panino could also achieve due to the presence of an integrase. Moreover, the presence of a phage copy of the tmRNA gene could serve to circumvent the inactivation of the host gene after insertion [69]. Sequencing of genomic DNA from virocells revealed no evidence of phage maintenance or integration, in solid and liquid nutrient rich media. However, it has been shown that under nutrient rich conditions [9] and high cell densities [70] phages prefer a lytic lifestyle. Previously identified phages infecting sponge symbionts also had similar ambiguous patterns of phage lifestyle. The alphaproteobacterial phage JL001 was shown to be temperate yet did not integrate into the host genome [60]. Due to the presence of two depolymerases with hydrolase activity, and no significant antiCRISPR protein homology, it is likely that host tropism is determined based on bacterial capsid composition [71], as *M. halichondriae* also doesn’t contain any known CRISPR sequences [54]. Therefore, a pseudolysogenic lifestyle potentially provides some competitive advantage for phages in the sponge microenvironment, and by extension to those sponge bacterial symbionts, due to extended maintenance under unfavorable conditions in the microhabitat [72]. Based on our results and findings of phages with similar lifestyle patterns, we suggest that phage Panino could have a pseudolysogenic lifestyle.

### Experimental perturbation of the sponge microbiome by phage application

To assess how the different groups within the sponge-associated microbiome respond to phage predation, we tested different modes of application of phage particles to the sponge that were used previously, i.e., incubation versus direct injection into the tissue [51, 73]. We assumed that direct delivery methods (injection) would have the largest impact on the communities in terms of culturable abundance (CFU counts), community richness (alpha diversity) and dissimilarity (beta diversity). While CFU abundance increased upon exposure to phages in all treatments, injection with a lower dose had the highest impact (at 72 h) (Fig. 4, Tables S4 and S5). The effect on relative bacterial abundances and diversity was similar across all phage treatments. Based on these findings, we suggest that the method of delivery might not have a strong effect for these filter-feeding animals. However, in case experimental manipulations interfere with the filtration capacity of sponges, a direct delivery method such as injection might be advantageous to bypass the uptake of particles from the water.

Even though direct bacterial lysis and phage replication was not shown in the current experiment, we propose the following scenarios for the observed increase in CFU counts. Based on known phage effects from literature, phage lysis could lead to the increase of nutrients from lysed cells which other bacteria use as growth substrates [74–76]. Moreover, an elimination of microbiota participating in nutrient blocking [50] could free up physical and nutrient niche spaces that have become available to new colonization [77]. Furthermore, since the phage target *M. halichondriae* has demonstrated to form biofilms in solid and liquid culture [54], we propose that the large increase in CFU counts observed in phage treatments could be explained by the break up of polymicrobial biofilms that may contain polysaccharides, proteins, and/or DNA as additional nutrients. Since an extracellular matrix also allows cells to adhere to each other and a variety of surfaces [78, 79], potentially, the increase in CFU could be attributed to the release of cells encased in the biofilm due to a phage-induced seeding dispersal event [80].

Alpha diversity quantifies the diversity of microbial species within a single sample or environment, offering insights into the richness and evenness of species abundance. Complementing culture-based measurements of CFU abundance, culture-independent bacterial diversity based on amplicon sequence variants (ASVs) significantly decreased after the onset (24 h) of the experiment, but recovered (72 h) in the control without phage application. Interestingly, phage application had no significant effect on alpha diversity compared to the control, but showed a significant decrease of diversity throughout the duration of the experiment (Fig. 5, Table S7). These contrasting effects of community development are known from sponge microbiomes when their bacterial community is impacted by environmental factors like pollution [81] and heat stress [82]. This indicates that while the initial conditions in the experiment may limit community development, shown by lower ASV richness and how evenly rare taxa occur, they only weakly affect the overall community dynamics afterwards. The initial abiotic disturbance (sterile artificial seawater), coupled with the effect of phage application, thus led to a larger disturbance in the bacterial community evident by the further significant increase in diversity in phage treatments. Despite the fact that the chosen phage infects a specific target, these observed community-wide changes indicate the phage to alter potential cross-feeding [83] or mutualistic interactions in the resident bacterial community [84].

Beta diversity quantifies the variation in microbial community composition among different samples or environments, in terms of dissimilarity or similarity between samples or groups. Similarly to findings in the alpha diversity, time had the strongest effect on bacterial communities (Fig. 6B) at the beta diversity, and the phage application increased variation in the community profile at the end of the experiment (72 h). We found that bacterial communities were more similar at the beginning (0 and 24 h) of the experiment, regardless of treatment, while at the end of the experiment communities showed higher dispersion with phage application (Table S10). Significant differences were observed also between the individual phage treatments, but only after 72 h, where direct delivery (injection) of higher phage dose (high) had larger effects than more indirect treatments (suspension) with lower phage dose (low) compared to the control (Table S9). The sudden increase in dispersal (24 to 72 h) after phage treatment might indicate that the bacterial communities reach a “tipping point” after which rapid change sets in, accompanied by a high turnover in the most abundant taxa—*Ca.* Halichondribacter (53% at 24 h to 42% at 72 h) and *Vibrio* (0.4% at 24 h to 13% at 72 h) (Fig. 6A, Table S8). Experiments with longer observations could further address whether such rapid community shifts eventually result in dysbiotic microbiome states with an effect on animal health or recover to initial states.

Our assessments of alpha and beta diversity changes after experimental phage perturbation showed significant community-wide perturbations within 72 h. Future studies are needed to monitor phage dynamics in sponge microbiomes over longer timescales, such as in pelagic microbial communities [49], in order to better assess the impact of such perturbations in natural systems and whether the observed patterns persist. The control treatment also showed a similar, albeit not significant, pattern as the phage treatments, which is perhaps due to the increased abiotic stress of an environment with reduced nutrients and increased sterility. Certainly, the degree of observed phage-induced effects might seem more exacerbated under artificial conditions, despite used concentrations of 10^4^–10^6^ PFU/mL being within the natural range of total viral abundance in seawater [85]. While similar concentrations for a single phage are probably not encountered in nature, such experimental approaches are necessary to target difficult to manipulate marine host-microbe players and gradually improve our understanding of complex interaction networks [Pita et al. [73]; Schmittmann et al. [51]).

### Phage-disturbance aids the proliferation of opportunistic bacteria leading to dysbiosis

We identified an increase of ASVs belonging to the genus *Vibrio* to be the main effect of phage treatments (LDA = 5.82, FDR < 0.001), compared to the reduction in abundance of symbiotic bacteria (Fig. S5, Table S11). During the course of the experiment, the relative abundance of the genus *Vibrio* increased from 0 to 13% in the phage treatment (Fig. 6A, Table S8). However, absolute quantification of marker genes by qPCR, or viable cell count by selective media plating is needed to reliably confirm biomass increase due to the compositional nature of 16S amplicon data [86]. *Vibrio* can be found as a low abundant member (< 1% relative abundance based on amplicon 16S data) of the sponge-associated microbiome in nature and aquaculture, sporadically present within sponge individuals. In experimental conditions, it has been shown to be a particularly strong opportunistic colonizer when the native microbiome is disturbed. Under sterile conditions, artificial sterile seawater, and after the application of antibiotics, *Vibrio* and *Pseudoalteromonas* increase in relative abundance several fold [51]. This marked increase in *Vibrio* with time was correlated with an increase in relative abundance of genera *Colwellia*, *Pseudoalteromonas*, *Photobacterium*, and *Shewanella* (Fig. S6), which likely exhibit similar opportunistic proliferation.

We’ve identified the main two ASVs of the genus *Vibrio* to potentially belong to *V. lentus*, *V. cyclotrophicus*, and *V. splendidus* (Table S14), known as marine bacteria that share a common trait as opportunistic colonizers and potential pathogens in marine animals. Those particular ASVs have been identified in diseased/decaying sponge individuals in aquaculture (data not shown) and upon antibiotic treatment [51]. These *Vibrio* species are well-adapted to marine environments and often reside in association with various marine organisms, ranging from plankton to fish and shellfish. These strains were reported to exploit compromised or weakened host defenses to establish infections, particularly in aquaculture settings where high-density farming can stress aquatic animals. *V. lentus* is known for causing infections in fish and shellfish, leading to significant economic impacts in aquaculture [87]. *V. cyclotrophicus*, while less studied, has the potential to affect marine organisms within their nutrient-rich cyclonic eddy habitats [88]. *V. splendidus* is notably pathogenic to a variety of marine invertebrates, including bivalves, and has been implicated in mass mortality events in aquaculture [89]. Due to their increased abundance in the sponge-associated bacterial communities disturbed by phages, it is possible these *Vibrio* taxa could exhibit their pathogenicity and further influence community dynamics.

Since phage Panino specifically targets an *H. panicea* sponge-associated bacterial strain from the genus *Maribacter*, we also expected to observe a decreasing trend in relative abundance in the corresponding genus. However, we did not find taxa of the genus *Maribacter* among the significantly differentially abundant signatures, when comparing the control and phage treatments at respective time points (Table S11, LDA = 4.56, FDR > 0.05), or a significant difference in abundance in the control without phage predation and the phage treatment (Fig. 6C). Since taxa of the genus *Maribacter* constitute < 1% relative abundance in the *H. panicea* 16S microbial profile, sensitivity of applied methods may be too low to reliably detect changes in relative abundance which may be further obfuscated by interindividual sponge variability [51]. The abundance pattern significantly correlated with changes in other sponge-associated taxa in the genera *Anderseniella* and *Kangiella* (Fig. S6), for which culturable and sponge-specific strains have been previously described [90, 91] and might be ecologically dependent on *M. halichondriae* within a polymicrobial biofilm [92].

**Fig. 7:**
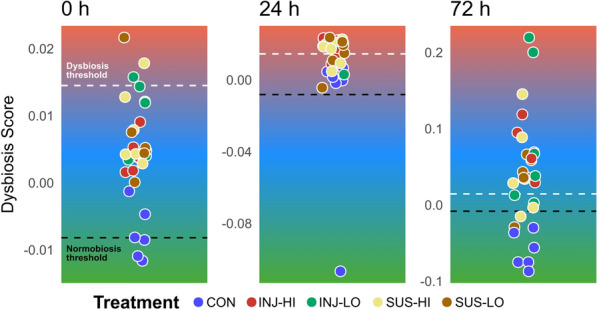
Distribution of dysbiosis scores as a measure of community divergence. Jitter plots based on Bray–Curtis dissimilarity and difference in Euclidian distances of each sample to the control as reference and their respective phage treatment group centroid at each time point (0, 24, 72 h). Thresholds of normobiosis and dysbiosis defined at time point 0 h as the 10th and 90th percentile. Control treatment (CON), injection into sponge tissue with high (INJ-HI) and low phage concentration (INJ-LO), suspension into seawater of high (SUS-HI) and low phage concentration (SUS-LO) treatments

Our assessment of bacterial community change and dysbiosis (Fig. 7), indicates a progressive increase of disturbance in the phage-treated groups over the duration of the experiment. Initially, 3/24 phage-treated samples were marked as potentially dysbiotic at 0 h, which increased to 16/24 at 24 h, and further to 19/24 at 72 h. This trend suggests that phage treatment, irrespective of concentration and mode of application, induces perturbations in the sponge microbiome over time, which could eventually lead to an altered or dysbiotic state associated with the invasion of opportunistic colonizers. Previously, sea star wasting disease has demonstrated the critical role of microbiome dysbiosis in marine animals, highlighting the proliferation of *Vibrio* in association with severe health decline [93]. The study illustrated that microbial imbalances are closely linked to disease outbreaks, as the dysbiotic state fosters conditions favorable to pathogenic and opportunistic microbes. This is supported by additional research showing that microbial dysbiosis precedes the signs of sea star wasting disease in wild populations of *Pisaster ochraceus*, and that microbiome shifts occur with the onset and progression of the disease [94]. Such findings emphasize the necessity of maintaining microbial stability to ensure the health of marine holobionts and prevent widespread disease. The higher dysbiosis scores observed at later time points in our perturbation experiment indicate a greater deviation from normobiosis, highlighting the disruptive impact of phage application on bacterial community structure and could potentially facilitate disease progression.

Phage effects are known to be exacerbated by the presence of bacteriocins [95]. *Vibrio*, but also Flavobacteriia like *Maribacter*, are known bacteriocin producers in marine animal-associated bacterial communities [96]. Vibrios are also known to be able to actively evade the effect of antimicrobial peptides (AMPs) which include bacteriocins [97]. Due to potential phage lysis of bacteriocin producers, additional bacteriocins are released into the microbiome environment, which is then taken advantage of by *Vibrio* since they have evolved the capacity to evade or resist potent AMPs. Such a scenario could explain the faster onset (24 h in phage treatments and 72 h in the control) of *Vibrio* proliferation (Fig. 6C), as other bacteriocin-sensitive taxa also diminish in abundance. As an alternative explanation, the release of nutrients into the environment by phage lysis is known to induce bacterial growth and increase bacterial biodiversity [6], which could explain proliferation of opportunists like *Vibrio* in the phage treatment. However, causal inferences of the plausible mechanisms of *Vibrio* dominance and the cascading effects of intra- and interspecies interactions within complex bacterial communities are difficult to address with correlative abundance patterns alone. Nonetheless, this study highlights the first demonstration of experimental microbiome manipulation in sponges, and gives insight into potential effects of single bacteriophages in complex marine host-associated environments in nature. Moreover, it confirms the potential of phage application in marine systems, in order to investigate the functional role and importance of specific microbiota by targeted elimination in sponge holobionts, or even in the long run, combat diseases caused by bacterial pathogens.

## Conclusion

In this study, we provide the first comprehensive assessment of a sponge-associated microbiome under phage disturbance. Together with previous findings from sponges and other model systems, these new results suggest that disturbance dynamics induced by single bacteriophages can have measurable effects when examined in a controlled experimental setting. We show that phage-induced disturbance can shift the scales in favor of opportunistic bacterial proliferation. Our study highlights the importance of experimental model systems that allow targeted manipulation in ecologically relevant marine organisms if we want to understand the processes governing microbiome assembly and holobiont homeostasis. Complementing our findings with additional experiments aiming at the targeted manipulation with other phages, with specific and broader host ranges, will allow us to understand the permanence of perturbations and long-term resilience of host-associated microbiomes. It remains to be seen whether such changes also occur at similar magnitudes and consequences in nature, or a type of buffering exists where co-occurrent phage-induced changes cancel each other out. Importantly, future studies will benefit from the integration of functional information and genome-wide sampling by means of metagenomics, and viromics for better viral resolution. With our experimental system, we introduce a novel approach to investigate the malleability of the sponge holobiont in response to phage-induced changes.

## Methods

### Sponge collection

*Halichondria panicea* individuals were collected by snorkeling in late September 2020 (phage isolation from sponge tissue) from Kiel, Germany (54.424705 N, 10.175133 E), and by SCUBA diving in early December 2020 (phage disturbance experiment) from Kiel, Germany (54.329899 N, 10.149413 E). Sponges with surrounding seawater were individually transported in 500 mL Kautex containers on ice and brought to Baltic flow-through tanks at the institute within 2 h after collection, for a 2-week acclimation period prior to experiments. Sponges used for phage isolation were immediately processed within the same day.

### Bacteria culturing

*Maribacter halichondriae* was cultured at 25 °C using Marine Broth (MB) medium (Marine Broth 2216, Becton Dickinson and Company, New Jersey, USA) either in aerated liquid culture (shaken at 120 rpm) or on 1.5% agar plates [54]. Bacterial stocks were frozen at − 20 °C with the Cryobank System (Mast Diagnostica GmbH, Reinfeld, Germany) and the bacterium was passaged twice on agar plates before use.

### Phage isolation

Viral particles within sponge tissue were extracted using a centrifugation-filtration method adapted from previous protocols on cultivation of phages from environmental samples and animal tissue [25, 98, 99]. Briefly, sponges were rinsed in sterile artificial seawater and the excess liquid was removed by dabbing with autoclaved tissue paper. A total of 500 g of sponge tissue was processed in 500 mL of seaweed extraction buffer (0.1 M Tris base, 0.1 M KCl, 52 mM MgSO_4_, 0.4 M NaCl, 10 mM CaCl_2_, 0.01 M Na_2_SO_3_, pH 7.6). Smaller aliquots of tissue and buffer in 50 mL Falcon tubes (Fisher Scientific, Hampton, NH, USA) were homogenized on ice with a blender at 6500 rpm (T25 digital ULTRA-TURRAX, IKA). Processed aliquots were combined again, and the crude homogenate was filtered through a paper filter, centrifuged at 2500 × *g* for 10 min and the supernatant was filtered through a 0.45 μm syringe filter (Sigma-Aldrich, St. Louis, MI, USA). The filtrate was then centrifuged at 2500 × *g* for 5 min and the supernatant filtered through a 0.22 μm syringe filter. Viral particles were precipitated using a 6% v/v final concentration of polyethylene glycol (PEG) 8000 (Sigma-Aldrich) and incubated at 4 °C for 2 h. The virus-PEG solution was centrifuged at 76000 × *g* at 4 °C for 30 min. The supernatant was discarded, and viral pellets were resuspended in 200 μL of 0.22 μm filtered SM-buffer (50 mM Tris–HCl,100 mM NaCl, 8 mM MgSO_4_ at pH 7.5). Finally, chloroform was added to a final concentration of 10% (v/v) to the phage solution and stored at 4 °C in the dark.

*M. halichondriae* was grown at room temperature until the exponential growth phase was reached. Serial dilutions of 100 μL of the viral extract were added to 900 μL of bacterial culture and incubated at room temperature for at least 15 min. Mixtures were then added to 4 mL of MB soft agar (0.7% agar), briefly vortexed and poured onto MB agar plates (1.5% agar). Plates were incubated at room temperature in the dark until plaques were observed. Phage isolates were further purified by picking plaques with a pipette tip and transferred into 1 mL of an exponentially growing bacterial culture. Viruses in the supernatant were concentrated with 6% v/v PEG 8000 as described above for the viral extract. *M. halichondriae* was reinfected with this concentrated phage solution as above and plaques were re-purified. The purification process was repeated twice to obtain pure phage isolates, which were stored at 4 °C. All phage stock preparations were consistently treated with 10% (v/v) chloroform to remove bacterial contamination which showed no effect on phage infectivity.

### Transmission electron microscopy

From a phage stock (~ 10^9^ PFU/mL), prepared from flooding confluent lysis or “webbed” plates with SM buffer, approximately 5 µL was used for morphological analysis using negative staining [100]. The samples were stained with 0.5% (wt/vol) aqueous uranyl acetate (Thermo Fisher Scientific, USA) and observed under a transmission electron microscope (FEI Tecnai G2 Spirit BioTWIN, Thermo Fisher Scientific, USA) operating at 80 kV, with magnifications ranging from 40,000× to 100,000×.

### Phage DNA extraction and genome sequencing

Genomic DNA from concentrated and purified viral particles was isolated with the Wizard DNA extraction kit (Promega) per the manufacturer’s instructions. Sequencing was done on the MinION (Oxford Nanopore Technologies, Oxford, UK) using a MinION Flongle Flow-Cell (Cat.No. FLO-FLG001) with the Flow Cell Priming Kit (Cat.No. EXP-FLP002) and the Rapid Sequencing Kit (Cat.No. SQK-RAD004), following the manufacturer protocols.

### Phage genome assembly, annotation and analysis

Basecalled, quality filtered and adapter-trimmed reads were used for long-read genome assembly with Canu [101]. The assembled genome was checked for quality before gene calling and annotation with PHANOTATE [102] and multiple bacterial and phage-specific databases. A circular genome map was drawn and labeled with best identified gene features. The phylogenetic placement and taxonomy were inferred by comparison with other related and previously published phages by VipTree [103] and genomic similarity calculated with VIRIDIC [104] (see supplementary methods file for more details).

### Phage host range test

Concentrated phage solution was spotted on top of overlay agar plates containing different bacterial strains from the genera *Maribacter*, *Aquimarina*, *Ruegeria*, *Shewanella*, *Limimaricola*, *Defluviimonas*, *Albidovulum*, *Frigidibacter*, *Paracoccus*, *Pseudorhodobacter*, and *Cellulophaga* isolated from *H. panicea* sponges and other marine sources. The plates were incubated at RT for 7 days and inspected for plaque formation every 24 h.

A 16S rRNA phylogenetic tree was created from tested isolates (see supplementary methods file for more details). Detailed list of all tested bacterial strains, taxonomic identity, culture collection ID, and 16S rRNA gene sequence Genbank ID is shown in Table S3.

### Experimental conditions

Experiments were performed in sterile marine chambers described previously [51]. A detailed description of the setup and materials used is provided on the online platform protocols.io [105], with the exception of antibiotic use and recolonization with a natural sponge microbiome. Briefly, we used multiple sterile 500 mL glass beakers, individually connected to aquarium pumps (GHL Doser2, GHL, Germany) for water exchange (twice the volume per day, 10 mL every 15 min) with sterile artificial seawater. Artificial seawater (Classic, Tropic Marin, Germany) was sterile filtered with a 0.22 μm membrane (Sartorius SM 162 75, 142 mm, Sartorius Stedim Biotech GmbH, Germany) into autoclaved 20 L carboys (Nalgene) and UV treated by passing through an aquarium UV light (Reeflex UV 2000, EHEIM, Germany) for 24 h. Fresh artificial seawater was prepared every day for the duration of the phage disturbance experiment. Experiments were performed at stable temperatures and salinities according to Baltic environmental conditions at the time of the experiment (12 °C, 16 PSU).

The purified phage isolate was enumerated by PFU plating [106] with the propagation host *M. halichondriae* one week before the beginning of the experiment. Two phage inocula were prepared from the enumerated stock by dilution with MB medium to ~ 10^9^ and ~ 10^7^ PFU. Phage Panino showed greatest stability when particles were suspended in MB medium, which is why it was used as the solvent of choice.

For the assessment of different phage application methods, a combination of two different phage particle delivery treatments—syringe injection into the sponge tissue (INJ) and resuspension into seawater surrounding the sponge (SUS), were tested in combination with two different doses of phage particles in the inoculum: ~ 10^9^ PFU defined as the high phage dose (HI) and ~ 10^7^ PFU as the low phage dose (LO). After dilution in 500 mL of seawater inside the beaker, the final concentration of phages yielded concentrations of ~ 10^6^ PFU/mL for HI and ~ 10^4^ PFU/mL for LO treatments. In total, four different combinations of phage application methods were tested: injection-high (INJ-HI), injection-low (INJ-LO), suspension-high (SUS-HI), and suspension-low (SUS-LO); along with a control treatment (CON) which contained no phage particles and consisted of a mock injection with MB media.

Six sponge individuals maintained in our Baltic aquaculture system with natural seawater from the Kiel fjord, were cut with scalpels and divided into 5 approximately equally sized explants (4 × 4 × 4 cm), and left to reequilibrate for two weeks before the start of the experiment. Afterwards, explants were transferred to sterile marine chambers, washed in artificial seawater, and placed in glass flow-through beakers prefilled with artificial seawater. Each explant from a single sponge individual was assigned to one of the four phage or the control treatments, and all beakers with explants were randomized within the chamber to minimize potential biases.

Sponge microbiome samples were obtained by repeated biopsy sampling at 0 (start), 24, and 72 h (end) of the experiment, after which the entire animal was sacrificed. Tissue samples for nucleic acid measurements were weighed, snap frozen in liquid nitrogen, and stored in − 80 °C. Tissue samples for direct plating of total culturable bacteria on MB agar plates were weighed, homogenized in sterile artificial seawater. Serial dilutions were prepared with liquid MB medium and three dilutions were plated out. Plates were incubated at 23 °C and total culturable CFU counts were obtained for each dilution in triplicate after 7 days (see supplementary methods file for details of statistical analyses).

### Microbiome DNA extraction

DNA was extracted from < 100 mg sponge tissue with the DNeasy PowerSoil Kit (Qiagen, Netherlands). The DNA was eluted in 50 μL elution buffer, quantified by Qubit (DNA BR and HS Kits, Thermo Fisher Scientific, USA) and purity checked with NanoDrop 2000c (Thermo Fisher Scientific, USA) [107].

### Amplicon sequencing and analysis

The V3-V4 variable regions of the 16S rRNA gene were amplified in a one-step PCR using the primer pair 341F-806R (dual-barcoding approach [108]; primer sequences: 5’-CCTACGGGAGG-CAGCAG-30 and 5’-GGACTACHVGGGTWTCTAAT-30). Paired-end sequencing (2 × 300 bp) was conducted on the MiSeq platform (Illumina, San Diego, USA) with v3 chemistry. The settings for demultiplexing were 0 mismatches in the barcode sequences.

Bioinformatic analyses followed a published protocol [109], were carried out within the QIIME2 environment [110], and all statistical analyses in R [111–113] (see supplementary methods file for more details). The dataset was analyzed as a whole (control vs phage), as well as subsets including control and separate phage treatments (control vs injection-high vs injection-low vs suspension-high vs suspension-low).

## Supplementary Information

Additional file 1. **Text S1** Supplementary text describing methods of bioinformatic analyses, computational tools and pipelines, and databases used.

Additional file 2. **Figure S1** Additional morphological and genomic features of Maribacter phage Panino. **(A)** Plaque assay of a serial stock dilution and development on a *M. halichondriae* lawn after 24, 48, and 72 h of incubation. **(B)** Distribution of tRNA gene counts in *Caudoviricetes* phage genomes (top), red arrow indicates the count of tRNAs in Maribacter phage Panino. Density distribution of tRNA to genome ratio in *Caudoviricetes* phage genomes (bottom), dashed line indicates the ratio value for Maribacter phage Panino.

Additional file 3. **Figure S2** Amplicon sequence variation (ASV) richness rarefaction curves based on species counts (Observed) and Shannon diversity index (Shannon). Each line shows a sponge sample within the treatment category with error bars corresponding to the standard deviation at subsampled depth intervals. **(A)** Rarefaction curves before subsampling with the lowest sample depth indicated with a black vertical line (5400 reads). **(B)** Rarefaction curves after subsampling at 5400 reads.

Additional file 4. **Figure S3** Alpha diversity measurement in sponge tissue between control (n = 6) and phage treatments (n = 24) over time. Boxplot comparison of species richness (observed count, Chao1 and Shannon’s diversity index) between control (CON) and phage-treated (PHI) samples at each time point (0, 24, 72 h). Data analyzed with a Kruskal–Wallis rank sum test, Dunn’s post-hoc test; comparisons were not statistically significant (p > 0.05).

Additional file 5. **Figure S4** Relative taxonomic abundance at the genus level of top 30 abundant taxa for all pooled samples of individual control (CON) and phage treatments (INJ-HI, INJ-LO, SUS-HI, SUS-LO) at 0, 24, and 72 h.

Additional file 6. **Figure S5** LDA scores of the top 30 differentially abundant significant taxa at the genus level in the bacterial community between control (CON) (n = 6) and phage (PHI) treatments (n = 24) at three time points (0, 24, 72 h), based on a Kruskal–Wallis rank-sum test (FDR < 0.05, LDA > 2.00). Heatmap indicates relative abundance classes between treatments for a given taxon (red—high, blue—low).

Additional file 7. **Figure S6** Pearson correlation barplot for the genus *Maribacter* and *Vibrio*, with 25 other genera. Bars indicate the value for the correlation coefficient of a taxon as positive (red) or negative correlations (blue) with darker colors (dark red, dark blue) indicating stronger correlation (R > 0.5). Heatmap indicates relative abundance classes between treatments for a given taxon (red—high, blue—low).

Additional file 8. **Table S1** List of functional features in the Maribacter phage Panino genome.

Additional file 9. **Table S2** List of related reference phages in the VipTree database with their genomic features, associated metadata, and similarity scores to Maribacter phage Panino.

Additional file 10. **Table S3** List of all bacterial strains used in the Maribacter phage Panino host range test, from publicly available and the private *H. panicea* strain collection, with associated metadata and 16S rRNA nucleotide sequence accessions.

Additional file 11. **Table S4** Results of the two-way Analysis of Variance (ANOVA), Linear Mixed Model (LMM), and individual linear regression models, testing the effect of time and individual phage treatments on culturable abundance (CFU counts).

Additional file 12. **Table S5** Results of the within-treatment one-way repeated measures ANOVA and between-treatment Welch's one-way ANOVA for effects of time and individual phage treatments on culturable abundance (CFU counts).

Additional file 13. **Table S6** Results of the within-treatment repeated measures Friedman rank sum test and between-treatment Wilcoxon signed-rank test for effects of time and individual phage treatments on alpha diversity (Chao1 index).

Additional file 14. **Table S7** Results of the within-treatment repeated measures Friedman rank sum test and between-treatment Kruskal–Wallis one-way ANOVA test for effects of time and grouped phage treatments on alpha diversity measures (Count, Chao1, Shannon).

Additional file 15. **Table S8** Taxonomic relative abundance table for control and grouped phage treatments at 0, 24, and 72 h at the family and genus level.

Additional file 16. **Table S9** Results of a PERMANOVA on beta diversity (Jensen﻿–Shannon dissimilarity) for control and individual phage treatments at 0, 24, and 72 h.

Additional file 17. **Table S10** Results of a PERMANOVA on beta diversity measures (Bray–Curtis, Jensen﻿–Shannon, Jaccard, weighted and unweighted Unifrac distances) for control and grouped phage treatments at 0, 24, and 72 h.

Additional file 18. **Table S11** Results of a LDA effect size analysis on taxa at the genus and ASV level for control and grouped phage treatments at 0, 24, and 72 h.

Additional file 19. **Table S12** Results of a single factor analysis with a Kruskal–Wallis test for control and grouped phage treatments at 72 h of relative abundances at the genus level.

Additional file 20. **Table S13** Results of a multiple linear regression analysis with MaAsLin2 for control and grouped phage treatments on taxa grouped at the genus level.

Additional file 21. **Table S14** BLAST analysis table for the identification of individual ASVs within the genus *Vibrio* (ordered in descending abundance) with their hits in the NCBI 16S rRNA database.

## Data Availability

Phage genome nucleotide sequence and annotation are available in the National Center for Biotechnology Information (NCBI) database Genbank: PP540027. All 16S rRNA gene amplicon reads, sample metadata and attributes are available in the NCBI Sequence Read Archive (SRA): PRJNA1003516; BioSample accessions: SAMN36892443-SAMN36892532.
