# Supplementary material for: Phage-induced disturbance of a marine sponge microbiome
Source: Environ Microbiome. 2024 Nov 26;19:97. doi: 10.1186/s40793-024-00637-7 (PMC11590407; doi:10.1186/s40793-024-00637-7)
Supplement: Supplementary file 1 — Additional file 1. Text S1 Supplementary text describing methods of bioinformatic analyses, computational tools and pipelines, and databases used. [file 40793_2024_637_MOESM1_ESM.docx]

# Bioinformatic methods

## Phage genome assembly, annotation, and analysis

The super-accurate model of Guppy (Oxford Nanopore Technologies plc. Version 6.2.1+6588110, dna_r9.4.1_450bps_sup) was used for basecalling of the MinION reads. Initially, the MinION data were assembled using Canu (v2.2) [[1]](https://paperpile.com/c/fuYRe0/wN9jl), then polished with Medaka (version 1.4.3, model r941_min_sup_g507, [[2]](https://paperpile.com/c/fuYRe0/TMWtr). Completeness and quality of all assemblies was assessed with CheckV [[3]](https://paperpile.com/c/fuYRe0/xc7ds). The final assembly (checkv_quality: Complete; miuvig_quality: High-quality; completeness: 100; completeness_method: DTR (high-confidence); contamination: 0; kmer_freq: 1) was checked for DTRs (1-234 bp, 77161-77394 bp) and the terminal redundancy confirmed by mapping reads back to the assembly with minimap2 [[4]](https://paperpile.com/c/fuYRe0/PG2yn). One set of redundancies (1-234 bp) was removed, yielding a final assembly length of 77160 bp with one redundancy retained at the end (76926-77160 bp) [[5, 6]](https://paperpile.com/c/fuYRe0/Uiugo+RhYGD).

Open reading frame (ORF) prediction and functional annotation of phage Panino was done using a combination of different gene finders - PHANOTATE [[7]](https://paperpile.com/c/fuYRe0/ZX5q2) (pharokka [[8]](https://paperpile.com/c/fuYRe0/AMlQR)), Prodigal [[9]](https://paperpile.com/c/fuYRe0/ORf8W) (Prokka [[10]](https://paperpile.com/c/fuYRe0/Q8dJ9), Bakta [[11]](https://paperpile.com/c/fuYRe0/VZC4v)), GeneMarkS [[12]](https://paperpile.com/c/fuYRe0/pacxe) (RAST [[11]](https://paperpile.com/c/fuYRe0/VZC4v)), and Balrog [[13]](https://paperpile.com/c/fuYRe0/mx8OE). Additional ORFs were predicted, manually inspected and curated with UGENE [[14]](https://paperpile.com/c/fuYRe0/IDhrX). Consensus gene calls and best hit predicted protein similarity searches against PHROGs [[15]](https://paperpile.com/c/fuYRe0/MO58z), VOG (https://vogdb.org), eggNOG [[16]](https://paperpile.com/c/fuYRe0/0JlvC), PFAM [[17]](https://paperpile.com/c/fuYRe0/gDcDz), PhaLP [[18]](https://paperpile.com/c/fuYRe0/vCZYj), and ACLAME [[19]](https://paperpile.com/c/fuYRe0/iZigt) databases were manually inspected and curated. Putative transfer RNA (tRNA) genes were identified using ARAGORN [[20]](https://paperpile.com/c/fuYRe0/tCtkl) and tRNAScan-SE [[21]](https://paperpile.com/c/fuYRe0/FNwll). Hypothetical proteins were further annotated by Bakta, DIAMOND [[22]](https://paperpile.com/c/fuYRe0/ec6mi) against the NCBI non-redundant (NR) protein database [[23]](https://paperpile.com/c/fuYRe0/uUsMa), PHASTER [[24]](https://paperpile.com/c/fuYRe0/UKr4h), PhaVIP [[25]](https://paperpile.com/c/fuYRe0/8v4Oz), PhageDPO [[26]](https://paperpile.com/c/fuYRe0/sirdg), STEP3 [[27]](https://paperpile.com/c/fuYRe0/sJv4y), VIRFAM [[28]](https://paperpile.com/c/fuYRe0/SmAQI), PaCRISPR [[29]](https://paperpile.com/c/fuYRe0/2M9GX), antiSMASH (v7.1.0) [[30]](https://paperpile.com/c/fuYRe0/PdsxL), and with HMMER3 [[31]](https://paperpile.com/c/fuYRe0/DvIOa) against Interpro [[32]](https://paperpile.com/c/fuYRe0/5DOAG) profiles of repL and ankyrin-repeat containing proteins. The hydrolase activity of putative depolymerases was further confirmed with PHYRE2 prediction [[33]](https://paperpile.com/c/fuYRe0/RDb8P). To compare the number of tRNA genes in tailed bacteriophage genomes, all *Caudoviricetes* phages were extracted from the INPHARED database (v1Nov2023) [[34]](https://paperpile.com/c/fuYRe0/3n3lx), subsetting a total of 17395 genomes plus phage Panino, and their tRNAs identified with tRNAScan-SE.

The graphical genome map was generated with the Proksee server tool [[35]](https://paperpile.com/c/fuYRe0/ZNobv) and genes grouped by PHROGs functional categories. The Panino genome was classified with other reference phages based on genome-wide similarities using VipTree (v4.0) [[36]](https://paperpile.com/c/fuYRe0/u9ywU). A proteomic tree was generated with the BIONJ algorithm, based on a genomic distance matrix, mid-point rooted, and a tree was regenerated by selecting the closest 19 reference phages according to their highest genomic similarity (S_G_) scores. Prokka was used for gene calling and protein sequences were added to the INPHARED protein database (v1Feb2024), which was used for taxonomic classification by guilt-by-contig-association with vConTACT2 [[37]](https://paperpile.com/c/fuYRe0/gxsbJ). Identification of nearest neighbors and reference genomes within viral clusters and network visualization was done with graphanalyzer [[38]](https://paperpile.com/c/fuYRe0/libat).

Intergenomic similarity with related phages identified by viral clustering is calculated for each viral genome, by alignment against all other genomes in the dataset using BLASTN with VIRIDIC [[39]](https://paperpile.com/c/fuYRe0/YlP5f). VIRIDIC generated heatmaps incorporate intergenomic similarity values (right half) and alignment indicators (left half and top annotation). In the right half, the color-coding shows the clustering of the phage genomes based on intergenomic similarity: the more closely-related the genomes, the darker the color (white to red). The numbers represent the similarity values for each genome pair, rounded to the first decimal. In the left half, three indicator values are represented for each genome pair, in the order from top to bottom: aligned fraction genome 1 (for the genome found in this row), genome length ratio (for the two genomes in this pair) and aligned fraction genome 2 (for the genome found in this column). The darker colors emphasize low values, indicating genome pairs where only a small fraction of the genome was aligned (blue to white color gradient), or where there is a high difference in the length of the two genomes (black to white color gradient). The aligned genome fractions are expected to decrease with increasing the distance between the phages. Therefore, darker colors should correspond to genome pairs with low similarity values, and whiter colors to genome pairs with higher similarity values. Similarly, more closely-related viruses are expected to have similar lengths.

## Bacterial 16S phylogenetic analysis

A 16S rRNA gene alignment was created from a subset of tested isolates in the host range test using SINA [[40]](https://paperpile.com/c/fuYRe0/ukFGN) with the SILVA 138 alignment [[41]](https://paperpile.com/c/fuYRe0/GQ8NY) as a reference. Maximum likelihood phylogenetic inference was performed using IQ-TREE web server (v1.6.12) [[42]](https://paperpile.com/c/fuYRe0/r685B). The best-fit model (TIMe+R16) for phylogenetic inference was selected based on Bayesian information criterion using ModelFinder [[43]](https://paperpile.com/c/fuYRe0/73IX7).

## Amplicon 16S analysis

Bioinformatic analyses followed a published protocol [[44]](https://paperpile.com/c/fuYRe0/ocNuO). For computation of microbial core-diversity metrics, sequences were processed within the QIIME2 environment (v2021.8) [[45]](https://paperpile.com/c/fuYRe0/5bgjk). Amplicon Sequence Variants (ASVs) were generated from forward reads (truncated to 270 nt) with the DADA2 algorithm [[46]](https://paperpile.com/c/fuYRe0/Z9RSk). Representative ASVs were classified using the Silva 138 99% OTUs 16S rRNA gene database [[41, 47]](https://paperpile.com/c/fuYRe0/GQ8NY+67lqJ) with the help of a primer-specific trained Naive Bayes taxonomic classifier. Mitochondrial, chloroplast and unassigned reads were removed. Rarefaction curves based on species counts (Observed richness) and the Shannon diversity index were constructed for all samples (Fig. S2). The data were rarefied to the sample with the lowest count, a sampling depth of 5400 reads per sample. Alpha and beta diversity indices were calculated within QIIME2, further microbiome analyses and visualizations were done with Phyloseq [[48]](https://paperpile.com/c/fuYRe0/wEMkF), MicrobiomeAnalyst [[49]](https://paperpile.com/c/fuYRe0/S6irW), ampvis2 [[50]](https://paperpile.com/c/fuYRe0/PT7lS), and vegan [[51]](https://paperpile.com/c/fuYRe0/PSCQu) R packages.

## Statistical analyses

### CFU count data

All statistical analyses were performed in R (v4.3.3) [[52]](https://paperpile.com/c/fuYRe0/XCHSy) and RStudio (v2023.12.1) [[53]](https://paperpile.com/c/fuYRe0/CDvCA) at a significance level of α = 0.05. For CFU count data, assumptions of normality were tested with the Shapiro-Wilk test (stats::shapiro.test). To assess the extent to which changes in time were associated with variations in CFU count a linear regression model was used (stats::lm), with CFU count as the dependent variable and time as the independent variable. Significant effects of time, control and phage treatments on CFU counts (n = 18) were tested with a two-way Analysis of Variance (ANOVA) (stats::aov; formula: CFU counts ~ Treatment * Time) and post-hoc pairwise comparisons were performed using the Tukey's Honestly Significant Difference test (stats::TukeyHSD). Additionally to account for correlations within individuals and across different time points we used a Linear Mixed Model (LMM) (nlme::nlm formula: CFU count ~ Treatment * Time, random = ~1 | Individual / Time) [[54]](https://paperpile.com/c/fuYRe0/WP15m) and post-hoc pairwise comparisons were done with the Tukey test in the {emmeans} package [[55]](https://paperpile.com/c/fuYRe0/2eCN7). Visual comparison and statistical testing within and between treatments were done with the {ggstatsplot} package [[56]](https://paperpile.com/c/fuYRe0/IqBdg), with functions grouped_ggwithinstats and grouped_ggbetweenstats. For within-treatment hypothesis testing we used a parametric test of a mixed effect model one-way repeated measures ANOVA, followed by a student's t-test for pairwise comparisons with Holm’s method for p-value adjustment. In between-treatment hypothesis testing we used a parametric test of Welch's one-way ANOVA (not assuming equal variances), followed by a Games-Howell test for pairwise comparisons with Holm’s method for p-value adjustment.

### Alpha diversity

Alpha diversity visual comparison and statistical testing within and between treatments were also done with the {ggstatsplot} package [[56]](https://paperpile.com/c/fuYRe0/IqBdg). For within-treatment hypothesis testing we used a non-parametric repeated measures Friedman rank sum test (n = 6), followed by a Durbin-Conover test for pairwise comparisons with Holm’s method for p-value adjustment. In between-treatment hypothesis testing we used a non-parametric Wilcoxon signed-rank test (n = 6), followed by a Dunn test for pairwise comparisons with Holm’s method for p-value adjustment.

Since individual phage treatments didn’t show significant differences between them in terms of alpha and beta diversity, we combined all the phage treatments and analyzed microbiome data as two groups - control (n = 6) and phage (n = 24). For grouped within-treatment hypothesis testing we used the same tests as for the individual treatment testing. In grouped between-treatment hypothesis testing we used a non-parametric Kruskal-Wallis one-way ANOVA test.

### Beta diversity

For beta diversity, ASV-level composition data (with and without removing singletons) were used to construct Bray-Curtis, Jensen-Shannon, Jaccard, weighted and unweighted Unifrac dissimilarity matrices, then PERMANOVA tests with 999 permutations were used to test for dissimilarity between treatments (individual (n = 6) and grouped phage treatments (n = 24)) at different timepoints. To evaluate sample separation in ordination space, a principal coordinate (PCoA) analysis was performed on Jensen-Shannon distances, and a regression analysis (stats::lm) was used to identify correlation of axes with experimental variables.

### Differential abundance

Univariate analyses of differential taxa abundances between control and phage treatments at the genus level were analyzed with the Kruskal-Wallis test with Benjamini–Hochberg method for false discovery rate (FDR) correction. Multiple linear regression with covariate adjustment analysis was carried out with MaAsLin2 [[57]](https://paperpile.com/c/fuYRe0/LX3wm) on taxa grouped at the genus level with cumulative sum scaling, log transformed, minimum abundance of 0.001 and minimum prevalence 0.1, standardized in respect to Time, with identity of individuals as random effects using a linear model, and Benjamini-Hochberg as FDR correction at a significance level of α = 0.05. Differential abundance analysis was performed using the Linear discriminant analysis Effect Size (LEfSe) method [[58]](https://paperpile.com/c/fuYRe0/IC5xI) also on taxa grouped at the genus level. LEfSe identifies features (taxa) that are differentially abundant between predefined groups while incorporating both statistical significance and biological relevance. Features with FDR < 0.05 and LDA > 2.0 were considered significant based on a Kruskal-Wallis rank-sum test at a significance level of α = 0.05. Correlation analyses of linear associations of genera *Maribacter* and *Vibrio* with other taxa at the genus level were done using Pearson’s correlation.

### Dysbiosis score

To contextualize the changes in community dissimilarity between phage treatments and their respective controls at different time points, we calculated a dysbiosis score based on Bray-Curtis dissimilarities [[59]](https://paperpile.com/c/fuYRe0/r499). For each sample, the differences of the euclidean distance to group centroids of a given reference and the distance to the tested group was calculated within dysbiosisR (v1.0.4) [[60]](https://paperpile.com/c/fuYRe0/R1Cw). At each time point, dysbiosis scores of samples in the phage treatments were calculated in reference to their samples in the control treatment. A dysbiosis score higher than 0 indicates more divergence from the reference group and potential dysbiosis, while scores of 0 indicate equal distance from the reference and test group. In addition, to capture changes in control and phage treatments over time, we defined thresholds of normobiosis at the 10th percentile of all samples at 0 h, before any significant effects of treatments occurred, and dysbiosis at the 90th percentile [[61]](https://paperpile.com/c/fuYRe0/m0vf).

# References

[1. Koren S, Walenz BP, Berlin K, Miller JR, Bergman NH, Phillippy AM. Canu: scalable and accurate long-read assembly via adaptive k-mer weighting and repeat separation. Genome Res. 2017;27:722–36.](http://paperpile.com/b/fuYRe0/wN9jl)

[2. Oxford Nanopore Technologies. medaka: Sequence correction provided by ONT Research. Github; 2017.](http://paperpile.com/b/fuYRe0/TMWtr)

[3. Nayfach S, Camargo AP, Schulz F, Eloe-Fadrosh E, Roux S, Kyrpides NC. CheckV assesses the quality and completeness of metagenome-assembled viral genomes. Nat Biotechnol. 2021;39:578–85.](http://paperpile.com/b/fuYRe0/xc7ds)

[4. Li H. New strategies to improve minimap2 alignment accuracy. Bioinformatics. 2021;37:4572–4.](http://paperpile.com/b/fuYRe0/PG2yn)

[5. Turner D, Adriaenssens EM, Tolstoy I, Kropinski AM. Phage Annotation Guide: Guidelines for Assembly and High-Quality Annotation. Phage (New Rochelle). 2021;2:170–82.](http://paperpile.com/b/fuYRe0/Uiugo)

[6. Shen A, Millard A. Phage Genome Annotation: Where to Begin and End. PHAGE. 2021;2:183–93.](http://paperpile.com/b/fuYRe0/RhYGD)

[7. McNair K, Zhou C, Dinsdale EA, Souza B, Edwards RA. PHANOTATE: a novel approach to gene identification in phage genomes. Bioinformatics. 2019;35:4537–42.](http://paperpile.com/b/fuYRe0/ZX5q2)

[8. Steinegger M, Söding J. MMseqs2 enables sensitive protein sequence searching for the analysis of massive data sets. Nat Biotechnol. 2017;35:1026–8.](http://paperpile.com/b/fuYRe0/AMlQR)

[9. Hyatt D, Chen G-L, Locascio PF, Land ML, Larimer FW, Hauser LJ. Prodigal: prokaryotic gene recognition and translation initiation site identification. BMC Bioinformatics. 2010;11:119.](http://paperpile.com/b/fuYRe0/ORf8W)

[10. Seemann T. Prokka: rapid prokaryotic genome annotation. Bioinformatics. 2014;30:2068–9.](http://paperpile.com/b/fuYRe0/Q8dJ9)

[11. Aziz RK, Bartels D, Best AA, DeJongh M, Disz T, Edwards RA, et al. The RAST Server: rapid annotations using subsystems technology. BMC Genomics. 2008;9:75.](http://paperpile.com/b/fuYRe0/VZC4v)

[12. Besemer J, Lomsadze A, Borodovsky M. GeneMarkS: a self-training method for prediction of gene starts in microbial genomes. Implications for finding sequence motifs in regulatory regions. Nucleic Acids Res. 2001;29:2607–18.](http://paperpile.com/b/fuYRe0/pacxe)

[13. Sommer MJ, Salzberg SL. Balrog: A universal protein model for prokaryotic gene prediction. PLoS Comput Biol. 2021;17:e1008727.](http://paperpile.com/b/fuYRe0/mx8OE)

[14. Rose R, Golosova O, Sukhomlinov D, Tiunov A, Prosperi M. Flexible design of multiple metagenomics classification pipelines with UGENE. Bioinformatics. 2019;35:1963–5.](http://paperpile.com/b/fuYRe0/IDhrX)

[15. Terzian P, Olo Ndela E, Galiez C, Lossouarn J, Pérez Bucio RE, Mom R, et al. PHROG: families of prokaryotic virus proteins clustered using remote homology. NAR Genom Bioinform. 2021;3:lqab067.](http://paperpile.com/b/fuYRe0/MO58z)

[16. Huerta-Cepas J, Szklarczyk D, Heller D, Hernández-Plaza A, Forslund SK, Cook H, et al. eggNOG 5.0: a hierarchical, functionally and phylogenetically annotated orthology resource based on 5090 organisms and 2502 viruses. Nucleic Acids Res. 2019;47:D309–14.](http://paperpile.com/b/fuYRe0/0JlvC)

[17. Mistry J, Chuguransky S, Williams L, Qureshi M, Salazar GA, Sonnhammer ELL, et al. Pfam: The protein families database in 2021. Nucleic Acids Res. 2021;49:D412–9.](http://paperpile.com/b/fuYRe0/gDcDz)

[18. Criel B, Taelman S, Van Criekinge W, Stock M, Briers Y. PhaLP: A Database for the Study of Phage Lytic Proteins and Their Evolution. Viruses. 2021;13.](http://paperpile.com/b/fuYRe0/vCZYj)

[19. Leplae R, Lima-Mendez G, Toussaint A. ACLAME: a CLAssification of Mobile genetic Elements, update 2010. Nucleic Acids Res. 2010;38 Database issue:D57–61.](http://paperpile.com/b/fuYRe0/iZigt)

[20. Laslett D, Canback B. ARAGORN, a program to detect tRNA genes and tmRNA genes in nucleotide sequences. Nucleic Acids Res. 2004;32:11–6.](http://paperpile.com/b/fuYRe0/tCtkl)

[21. Schattner P, Brooks AN, Lowe TM. The tRNAscan-SE, snoscan and snoGPS web servers for the detection of tRNAs and snoRNAs. Nucleic Acids Res. 2005;33 Web Server issue:W686–9.](http://paperpile.com/b/fuYRe0/FNwll)

[22. Buchfink B, Reuter K, Drost H-G. Sensitive protein alignments at tree-of-life scale using DIAMOND. Nat Methods. 2021;18:366–8.](http://paperpile.com/b/fuYRe0/ec6mi)

[23. Sayers EW, Bolton EE, Brister JR, Canese K, Chan J, Comeau DC, et al. Database resources of the national center for biotechnology information. Nucleic Acids Res. 2022;50:D20–6.](http://paperpile.com/b/fuYRe0/uUsMa)

[24. Arndt D, Grant JR, Marcu A, Sajed T, Pon A, Liang Y, et al. PHASTER: a better, faster version of the PHAST phage search tool. Nucleic Acids Res. 2016;44:W16–21.](http://paperpile.com/b/fuYRe0/UKr4h)

[25. Shang J, Peng C, Tang X, Sun Y. PhaVIP: Phage VIrion Protein classification based on chaos game representation and Vision Transformer. Bioinformatics. 2023;39 39 Suppl 1:i30–9.](http://paperpile.com/b/fuYRe0/8v4Oz)

[26. Vieira M, Duarte J, Domingues R, Oliveira H, Dias O. PhageDPO: Phage Depolymerase Finder. bioRxiv. 2023;:2023.02.24.529883.](http://paperpile.com/b/fuYRe0/sirdg)

[27. Thung TY, White ME, Dai W, Wilksch JJ, Bamert RS, Rocker A, et al. Component Parts of Bacteriophage Virions Accurately Defined by a Machine-Learning Approach Built on Evolutionary Features. mSystems. 2021;6:e0024221.](http://paperpile.com/b/fuYRe0/sJv4y)

[28. Lopes A, Tavares P, Petit M-A, Guérois R, Zinn-Justin S. Automated classification of tailed bacteriophages according to their neck organization. BMC Genomics. 2014;15:1027.](http://paperpile.com/b/fuYRe0/SmAQI)

[29. Wang J, Dai W, Li J, Xie R, Dunstan RA, Stubenrauch C, et al. PaCRISPR: a server for predicting and visualizing anti-CRISPR proteins. Nucleic Acids Res. 2020;48:W348–57.](http://paperpile.com/b/fuYRe0/2M9GX)

[30. Blin K, Shaw S, Augustijn HE, Reitz ZL, Biermann F, Alanjary M, et al. antiSMASH 7.0: new and improved predictions for detection, regulation, chemical structures and visualisation. Nucleic Acids Res. 2023;51:W46–50.](http://paperpile.com/b/fuYRe0/PdsxL)

[31. Finn RD, Clements J, Eddy SR. HMMER web server: interactive sequence similarity searching. Nucleic Acids Res. 2011;39 Web Server issue:W29–37.](http://paperpile.com/b/fuYRe0/DvIOa)

[32. Paysan-Lafosse T, Blum M, Chuguransky S, Grego T, Pinto BL, Salazar GA, et al. InterPro in 2022. Nucleic Acids Res. 2023;51:D418–27.](http://paperpile.com/b/fuYRe0/5DOAG)

[33. Kelley LA, Mezulis S, Yates CM, Wass MN, Sternberg MJE. The Phyre2 web portal for protein modeling, prediction and analysis. Nat Protoc. 2015;10:845–58.](http://paperpile.com/b/fuYRe0/RDb8P)

[34. Cook R, Brown N, Redgwell T, Rihtman B, Barnes M, Clokie M, et al. INfrastructure for a PHAge REference Database: Identification of Large-Scale Biases in the Current Collection of Cultured Phage Genomes. PHAGE. 2021;2:214–23.](http://paperpile.com/b/fuYRe0/3n3lx)

[35. Grant JR, Enns E, Marinier E, Mandal A, Herman EK, Chen C-Y, et al. Proksee: in-depth characterization and visualization of bacterial genomes. Nucleic Acids Res. 2023;51:W484–92.](http://paperpile.com/b/fuYRe0/ZNobv)

[36. Nishimura Y, Yoshida T, Kuronishi M, Uehara H, Ogata H, Goto S. ViPTree: the viral proteomic tree server. Bioinformatics. 2017;33:2379–80.](http://paperpile.com/b/fuYRe0/u9ywU)

[37. Bin Jang H, Bolduc B, Zablocki O, Kuhn JH, Roux S, Adriaenssens EM, et al. Taxonomic assignment of uncultivated prokaryotic virus genomes is enabled by gene-sharing networks. Nat Biotechnol. 2019;37:632–9.](http://paperpile.com/b/fuYRe0/gxsbJ)

[38. Pandolfo M, Telatin A, Lazzari G, Adriaenssens EM, Vitulo N. MetaPhage: an Automated Pipeline for Analyzing, Annotating, and Classifying Bacteriophages in Metagenomics Sequencing Data. mSystems. 2022;7:e0074122.](http://paperpile.com/b/fuYRe0/libat)

[39. Moraru C, Varsani A, Kropinski AM. VIRIDIC-A Novel Tool to Calculate the Intergenomic Similarities of Prokaryote-Infecting Viruses. Viruses. 2020;12.](http://paperpile.com/b/fuYRe0/YlP5f)

[40. Pruesse E, Peplies J, Glöckner FO. SINA: accurate high-throughput multiple sequence alignment of ribosomal RNA genes. Bioinformatics. 2012;28:1823–9.](http://paperpile.com/b/fuYRe0/ukFGN)

[41. Quast C, Pruesse E, Yilmaz P, Gerken J, Schweer T, Yarza P, et al. The SILVA ribosomal RNA gene database project: improved data processing and web-based tools. Nucleic Acids Res. 2013;41 Database issue:D590–6.](http://paperpile.com/b/fuYRe0/GQ8NY)

[42. Trifinopoulos J, Nguyen L-T, von Haeseler A, Minh BQ. W-IQ-TREE: a fast online phylogenetic tool for maximum likelihood analysis. Nucleic Acids Res. 2016;44:W232–5.](http://paperpile.com/b/fuYRe0/r685B)

[43. Kalyaanamoorthy S, Minh BQ, Wong TKF, von Haeseler A, Jermiin LS. ModelFinder: fast model selection for accurate phylogenetic estimates. Nat Methods. 2017;14:587–9.](http://paperpile.com/b/fuYRe0/73IX7)

[44. Busch K, Slaby BM, Bach W, Boetius A, Clefsen I, Colaço A, et al. Biodiversity, environmental drivers, and sustainability of the global deep-sea sponge microbiome. Nat Commun. 2022;13:5160.](http://paperpile.com/b/fuYRe0/ocNuO)

[45. Bolyen E, Rideout JR, Dillon MR, Bokulich NA, Abnet CC, Al-Ghalith GA, et al. Reproducible, interactive, scalable and extensible microbiome data science using QIIME 2. Nat Biotechnol. 2019;37:852–7.](http://paperpile.com/b/fuYRe0/5bgjk)

[46. Callahan BJ, McMurdie PJ, Rosen MJ, Han AW, Johnson AJA, Holmes SP. DADA2: High-resolution sample inference from Illumina amplicon data. Nat Methods. 2016;13:581–3.](http://paperpile.com/b/fuYRe0/Z9RSk)

[47. Robeson MS 2nd, O’Rourke DR, Kaehler BD, Ziemski M, Dillon MR, Foster JT, et al. RESCRIPt: Reproducible sequence taxonomy reference database management. PLoS Comput Biol. 2021;17:e1009581.](http://paperpile.com/b/fuYRe0/67lqJ)

[48. McMurdie PJ, Holmes S. phyloseq: an R package for reproducible interactive analysis and graphics of microbiome census data. PLoS One. 2013;8:e61217.](http://paperpile.com/b/fuYRe0/wEMkF)

[49. Dhariwal A, Chong J, Habib S, King IL, Agellon LB, Xia J. MicrobiomeAnalyst: a web-based tool for comprehensive statistical, visual and meta-analysis of microbiome data. Nucleic Acids Res. 2017;45:W180–8.](http://paperpile.com/b/fuYRe0/S6irW)

[50. Andersen KS, Kirkegaard RH, Karst SM, Albertsen M. ampvis2: an R package to analyse and visualise 16S rRNA amplicon data. bioRxiv. 2018;:299537.](http://paperpile.com/b/fuYRe0/PT7lS)

[51. Dixon P. VEGAN, a package of R functions for community ecology. J Veg Sci. 2003;14:927–30.](http://paperpile.com/b/fuYRe0/PSCQu)

[52. R Core Team. R: A Language and Environment for Statistical Computing. Vienna, Austria: R Foundation for Statistical Computing; 2024.](http://paperpile.com/b/fuYRe0/XCHSy)

[53. Posit team. RStudio: Integrated Development Environment for R. Boston, MA: Posit Software, PBC; 2024.](http://paperpile.com/b/fuYRe0/CDvCA)

[54. Pinheiro J, Bates D, R Core Team. nlme: Linear and Nonlinear Mixed Effects Models. 2023.](http://paperpile.com/b/fuYRe0/WP15m)

[55. Lenth RV. emmeans: Estimated Marginal Means, aka Least-Squares Means. 2024.](http://paperpile.com/b/fuYRe0/2eCN7)

[56. Patil I. Visualizations with statistical details: The “ggstatsplot” approach. J Open Source Softw. 2021;6:3167.](http://paperpile.com/b/fuYRe0/IqBdg)

[57. Mallick H, Rahnavard A, McIver LJ, Ma S, Zhang Y, Nguyen LH, et al. Multivariable association discovery in population-scale meta-omics studies. PLoS Comput Biol. 2021;17:e1009442.](http://paperpile.com/b/fuYRe0/LX3wm)

[58. Segata N, Izard J, Waldron L, Gevers D, Miropolsky L, Garrett WS, et al. Metagenomic biomarker discovery and explanation. Genome Biol. 2011;12:R60.](http://paperpile.com/b/fuYRe0/IC5xI)

[59. AlShawaqfeh MK, Wajid B, Minamoto Y, Markel M, Lidbury JA, Steiner JM, et al. A dysbiosis index to assess microbial changes in fecal samples of dogs with chronic inflammatory enteropathy. FEMS Microbiol Ecol. 2017;93:fix136.](http://paperpile.com/b/fuYRe0/r499)

[60. Wei S, Bahl MI, Baunwall SMD, Hvas CL, Licht TR. Determining gut microbial dysbiosis: A review of applied indexes for assessment of intestinal Microbiota imbalances. Appl Environ Microbiol. 2021;87.](http://paperpile.com/b/fuYRe0/R1Cw)

[61. Lloyd-Price J, Arze C, Ananthakrishnan AN, Schirmer M, Avila-Pacheco J, Poon TW, et al. Multi-omics of the gut microbial ecosystem in inflammatory bowel diseases. Nature. 2019;569:655–62.](http://paperpile.com/b/fuYRe0/m0vf)
