# Supplementary figures and images for: Phage-induced disturbance of a marine sponge microbiome
Source: Environ Microbiome. 2024 Nov 26;19:97. doi: 10.1186/s40793-024-00637-7 (PMC11590407; doi:10.1186/s40793-024-00637-7)

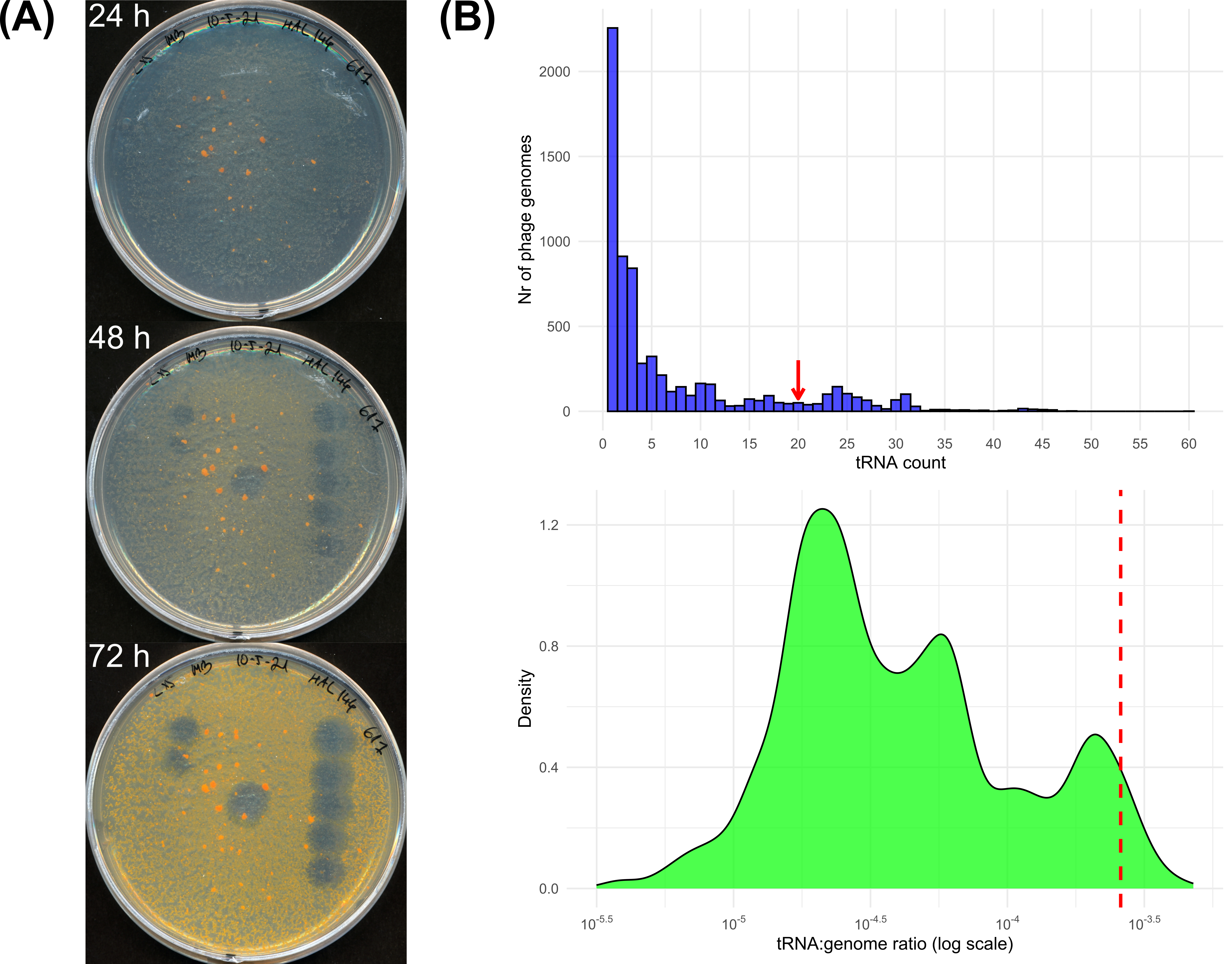

Supplement: Supplementary file 2 — Additional file 2. Figure S1 Additional morphological and genomic features of Maribacter phage Panino. (A) Plaque assay of a serial stock dilution and development on a M. halichondriae lawn after 24, 48, and 72 h of incubation. (B) Distribution of tRNA gene counts in Caudoviricetes phage genomes (top), red arrow indicates the count of tRNAs in Maribacter phage Panino. Density distribution of tRNA to genome ratio in Caudoviricetes phage genomes (bottom), dashed line indicates the ratio value for Maribacter phage Panino. [file 40793_2024_637_MOESM2_ESM.png]

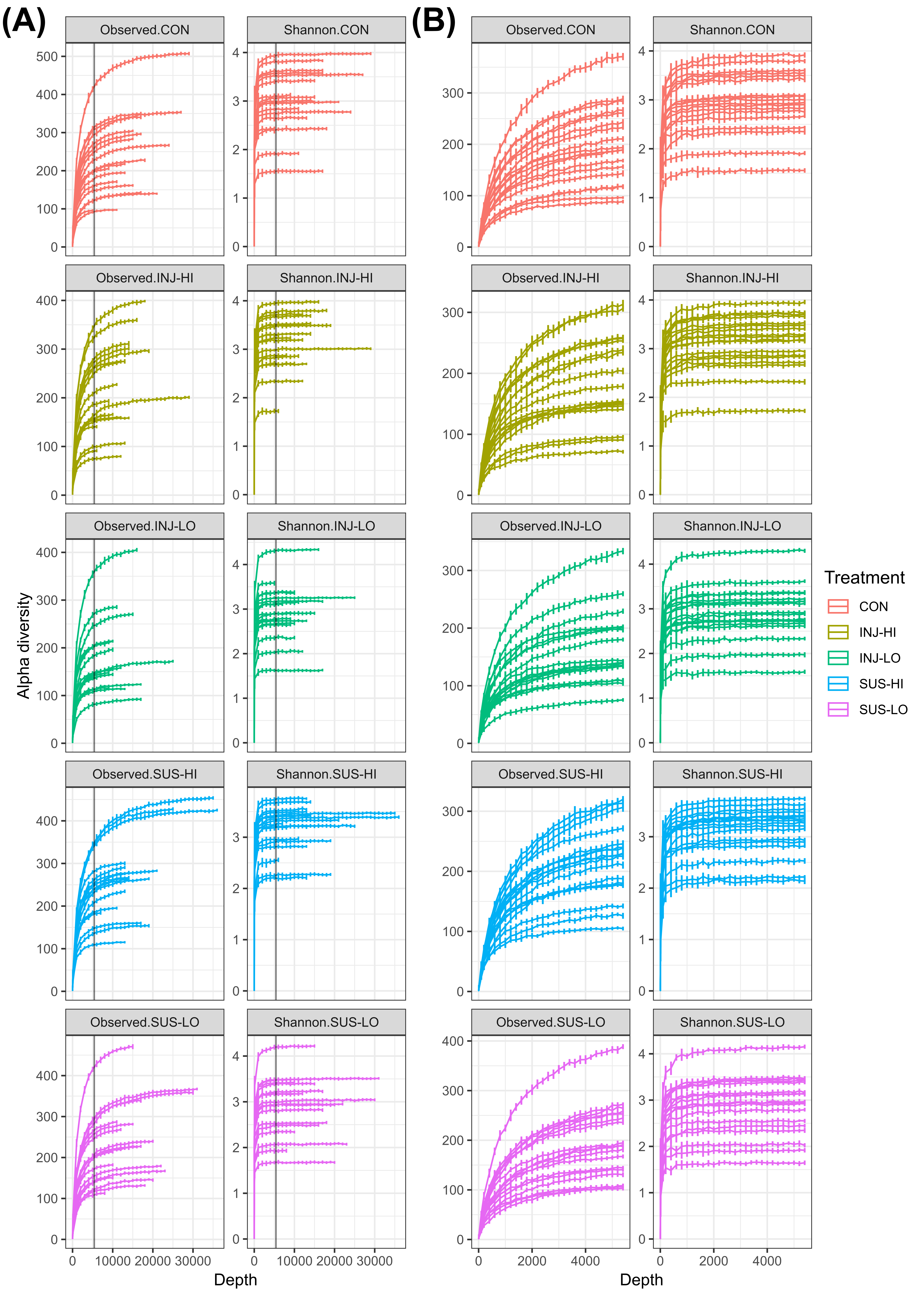

Supplement: Supplementary file 3 — Additional file 3. Figure S2 Amplicon sequence variation (ASV) richness rarefaction curves based on species counts (Observed) and Shannon diversity index (Shannon). Each line shows a sponge sample within the treatment category with error bars corresponding to the standard deviation at subsampled depth intervals. (A) Rarefaction curves before subsampling with the lowest sample depth indicated with a black vertical line (5400 reads). (B) Rarefaction curves after subsampling at 5400 reads. [file 40793_2024_637_MOESM3_ESM.png]

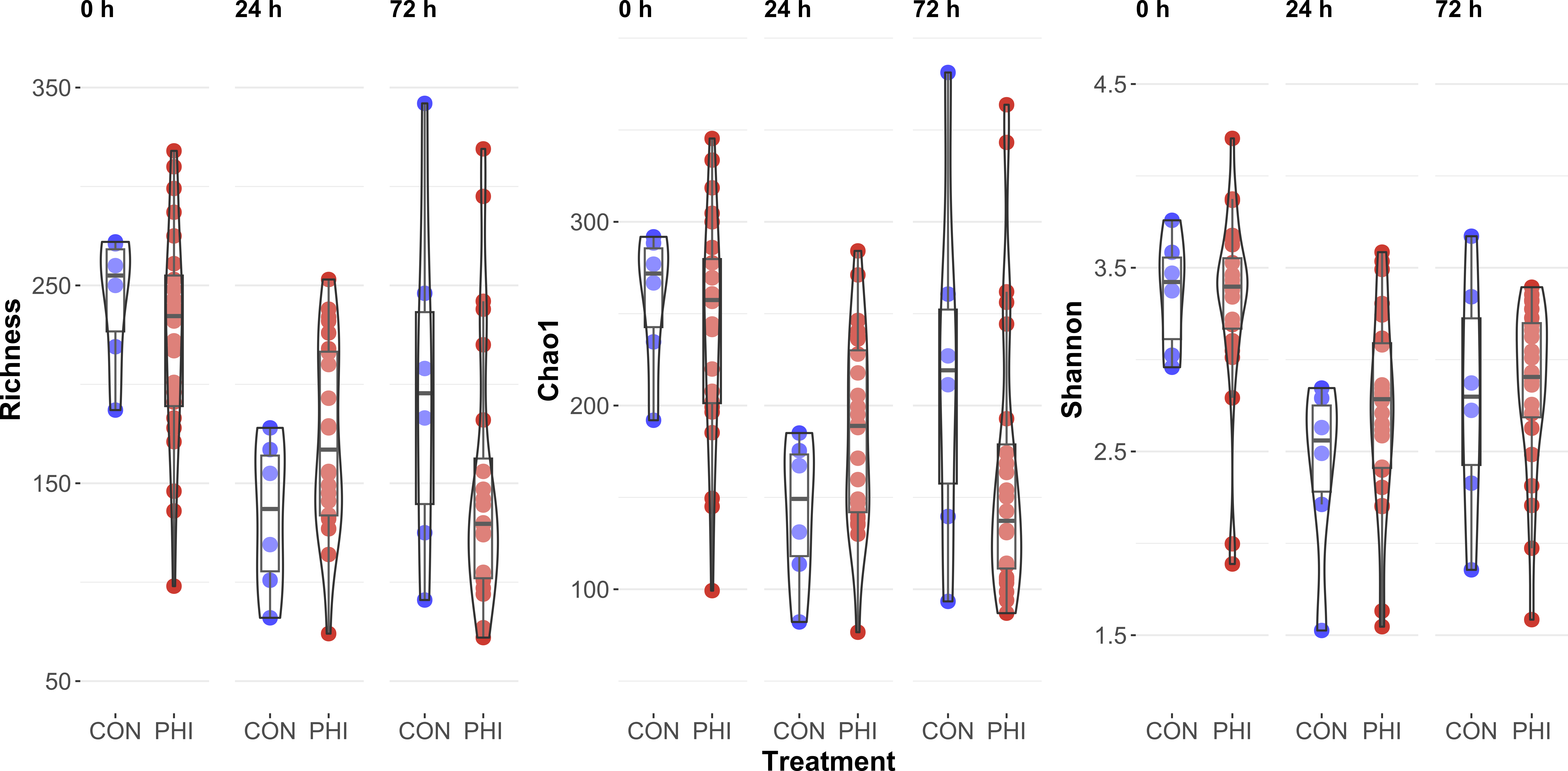

Supplement: Supplementary file 4 — Additional file 4. Figure S3 Alpha diversity measurement in sponge tissue between control (n = 6) and phage treatments (n = 24) over time. Boxplot comparison of species richness (observed count, Chao1 and Shannon’s diversity index) between control (CON) and phage-treated (PHI) samples at each time point (0, 24, 72 h). Data analyzed with a Kruskal–Wallis rank sum test, Dunn’s post-hoc test; comparisons were not statistically significant (p > 0.05). [file 40793_2024_637_MOESM4_ESM.png]

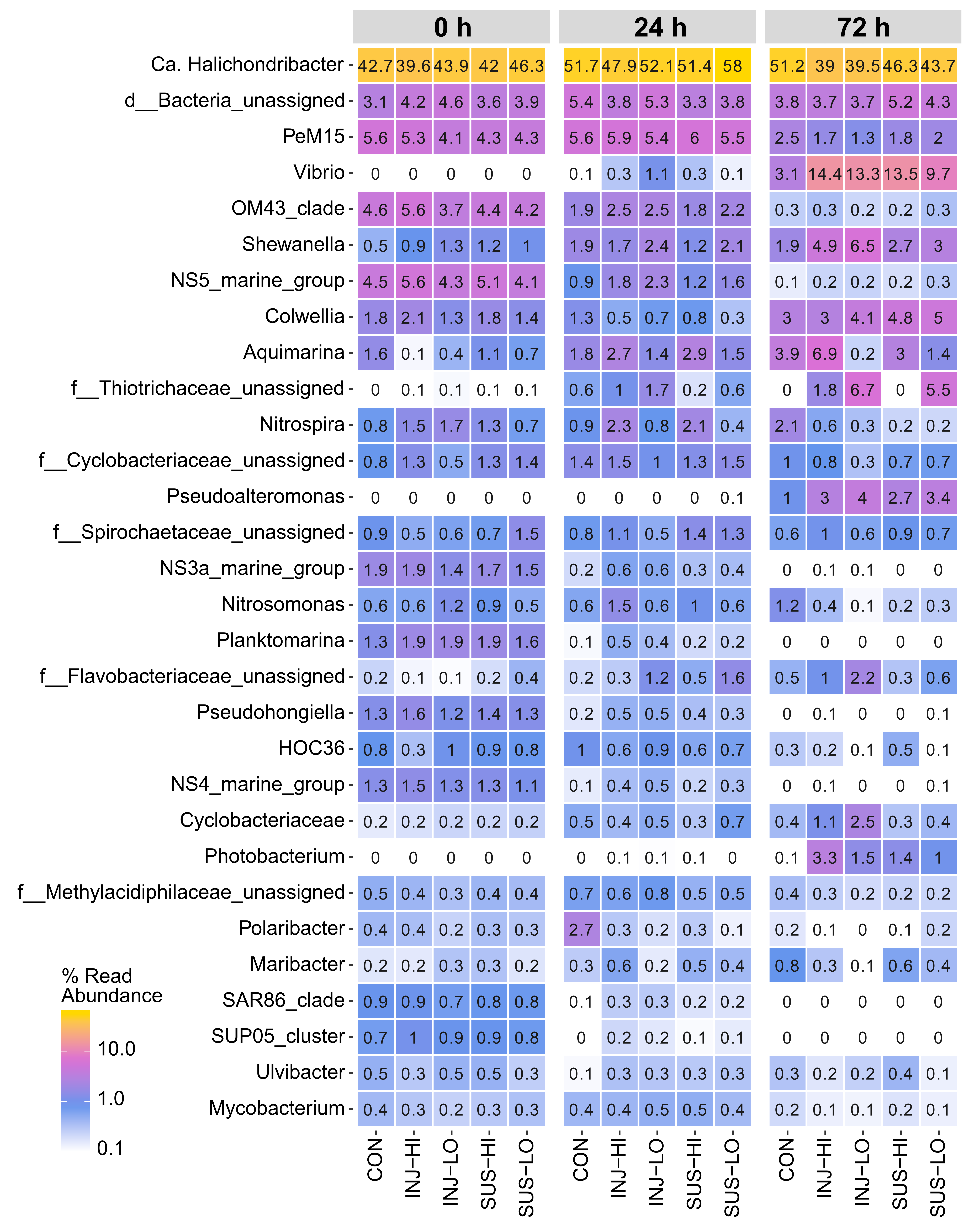

Supplement: Supplementary file 5 — Additional file 5. Figure S4 Relative taxonomic abundance at the genus level of top 30 abundant taxa for all pooled samples of individual control (CON) and phage treatments (INJ-HI, INJ-LO, SUS-HI, SUS-LO) at 0, 24, and 72 h. [file 40793_2024_637_MOESM5_ESM.png]
